# Association of Extubation Failure Rates With High-Flow Nasal Cannula, Continuous Positive Airway Pressure, and Bilevel Positive Airway Pressure vs Conventional Oxygen Therapy in Infants and Young Children: A Systematic Review and Network Meta-Analysis

**DOI:** 10.1001/jamapediatrics.2023.1478

**Published:** 2023-06-05

**Authors:** Narayan Prabhu Iyer, Alexandre T. Rotta, Sandrine Essouri, Jose Roberto Fioretto, Hannah J. Craven, Elizabeth C. Whipple, Padmanabhan Ramnarayan, Samer Abu-Sultaneh, Robinder G. Khemani

**Affiliations:** 1 Division of Neonatology, Fetal and Neonatal Institute, Children’s Hospital Los Angeles, Los Angeles, California; 2 Department of Pediatrics, Keck School of Medicine, University of Southern California, Los Angeles; 3 Department of Pediatrics, Division of Pediatric Critical Care Medicine, Duke University, Durham, North Carolina; 4 Department of Pediatrics, Sainte-Justine Hospital, Université de Montréal, Montreal, Quebec, Canada; 5 Department of Pediatrics, Pediatric Critical Care Division, Botucatu Medical School - UNESP-Sao Paulo State University, Botucatu, Sao Paulo, Brazil; 6 Ruth Lilly Medical Library, Indiana University School of Medicine, Indianapolis; 7 Faculty of Medicine, Department of Surgery and Cancer, Imperial College London, London, United Kingdom; 8 Department of Pediatrics, Division of Pediatric Critical Care, Riley Hospital for Children at Indiana University Health and Indiana University School of Medicine, Indianapolis; 9 Department of Anesthesiology and Critical Care, Children’s Hospital Los Angeles, Los Angeles, California; 10 Children’s Hospital Los Angeles, University of Southern California Keck School of Medicine, Los Angeles

## Abstract

**Importance:**

Extubation failure (EF) has been associated with worse outcomes in critically ill children. The relative efficacy of different modes of noninvasive respiratory support (NRS) to prevent EF is unknown.

**Objective:**

To study the reported relative efficacy of different modes of NRS (high-flow nasal cannula [HFNC], continuous positive airway pressure [CPAP], and bilevel positive airway pressure [BiPAP]) compared to conventional oxygen therapy (COT).

**Data Sources:**

MEDLINE, Embase, and CINAHL Complete through May 2022.

**Study Selection:**

Randomized clinical trials that enrolled critically ill children receiving invasive mechanical ventilation for more than 24 hours and compared the efficacy of different modes of postextubation NRS.

**Data Extraction and Synthesis:**

Random-effects models were fit using a bayesian network meta-analysis framework. Between-group comparisons were estimated using odds ratios (ORs) or mean differences with 95% credible intervals (CrIs). Treatment rankings were assessed by rank probabilities and the surface under the cumulative rank curve (SUCRA).

**Main Outcomes and Measures:**

The primary outcome was EF (reintubation within 48 to 72 hours). Secondary outcomes were treatment failure (TF, reintubation plus NRS escalation or crossover to another NRS mode), pediatric intensive care unit (PICU) mortality, PICU and hospital length of stay, abdominal distension, and nasal injury.

**Results:**

A total of 11 615 citations were screened, and 9 randomized clinical trials with a total of 1421 participants were included. Both CPAP and HFNC were found to be more effective than COT in reducing EF and TF (CPAP: OR for EF, 0.43; 95% CrI, 0.17-1.0 and OR for TF 0.27, 95% CrI 0.11-0.57 and HFNC: OR for EF, 0.64; 95% CrI, 0.24-1.0 and OR for TF, 0.34; 95% CrI, 0.16- 0.65). CPAP had the highest likelihood of being the best intervention for both EF (SUCRA, 0.83) and TF (SUCRA, 0.91). Although not statistically significant, BiPAP was likely to be better than COT for preventing both EF and TF. Compared to COT, CPAP and BiPAP were reported as showing a modest increase (approximately 3%) in nasal injury and abdominal distension.

**Conclusions and Relevance:**

The studies included in this systematic review and network meta-analysis found that compared with COT, EF and TF rates were lower with modest increases in abdominal distension and nasal injury. Of the modes evaluated, CPAP was associated with the lowest rates of EF and TF.

## Key Points

**Question:**

What is the most effective postextubation noninvasive respiratory support modality in children?

**Findings:**

In this systematic review and network meta-analysis, extubation failure and treatment failure rates were lower with continuous positive airway pressure (CPAP), high-flow nasal cannula (HFNC), and bilevel positive airway pressure (BiPAP) compared to conventional oxygen therapy (COT). Based on bayesian ranking probabilities, CPAP was reported to be the most effective of the evaluated noninvasive respiratory support modes for the prevention of extubation failure and treatment failure.

**Meaning:**

The results suggest that CPAP, HFNC, and BiPAP were more effective than COT for providing postextubation NRS in a pediatric population.

## Teaser

This systematic review and network meta-analysis evaluates 3 noninvasive respiratory support modes for preventing extubation failure in children compared with conventional oxygen therapy.

## Introduction

Extubation failure (EF) is an important event that is associated with poor clinical outcomes in pediatric intensive care units (PICUs).^1,2,3^ Postextubation noninvasive respiratory support (NRS), including high-flow nasal cannula (HFNC), continuous positive airway pressure (CPAP), and bilevel positive airway pressure (BiPAP), is frequently used in PICUs in an attempt to reduce the risk of EF. Several randomized clinical trials and observational studies have tried to evaluate the efficacy of various modes of NRS, but based on the current evidence, it is unclear whether NRS is superior to conventional oxygen therapy (COT) in preventing EF and which type of NRS is the most effective.

Pooling of evidence from randomized clinical trials and or observational studies using a meta-analytic model is considered the highest form of evidence.^4^ However, a standard pairwise meta-analysis is limited when there is a high degree of heterogeneity among studies, particularly when the interventions and comparators differ (ie, different forms of NRS). Therefore, we designed a systematic review and network meta-analysis to study the relative efficacy reported for different modes of NRS in preventing EF and other patient-centered outcomes among critically ill children.

## Methods

To prepare this report, we used the Preferred Reporting Items for Systematic Reviews and Meta-analyses (PRISMA) reporting guideline (eTable 1 in Supplement 1).^5^ This review was conducted as part of a project to develop clinical practice guidelines for ventilator liberation in children.^6,7^ The protocol for the systematic review is registered in PROSPERO (CRD42021228702). Details of the protocol for the systematic review can be accessed at https://www.crd.york.ac.uk/PROSPERO/display_record.php?ID=CRD42021228702.

### Population, Interventions, and Outcomes

This systematic review and network meta-analysis was designed to answer the following questions. In children who are hospitalized in the short term, is postextubation NRS more effective than COT in preventing EF? What is the reported relative efficacy of different modes of NRS in preventing EF? The population in included studies comprised critically ill children from birth (born at 37 weeks’ gestation or later) to age 18 years receiving invasive mechanical ventilation for more than 24 hours and being supported by postextubation NRS either as rescue or planned prior to extubation. The different modes of NRS included HFNC, CPAP, and BiPAP using any patient interface.

Outcomes were selected prior to the literature search and rated for their patient centeredness and importance using the Grading of Recommendations Assessment, Development and Evaluation (GRADE) approach.^8^ The panel of experts categorized the outcomes as follows. Critical outcomes included mortality, failure to liberate from invasive mechanical ventilation (ie, EF, ,defined as reintubation within 48 to 72 hours), PICU length of stay, escalation of care or crossover to other treatments, and treatment failure (TF; reintubation or escalation/crossover to another NRS mode). Important outcomes included liberation from NRS, total duration of NRS, number of ventilator-free days, hospital length of stay, and pressure injuries. One outcome of limited importance was included, namely, abdominal distension.

### Literature Search and Data Collection

Comprehensive search strategies were composed and conducted by 1 of 2 medical librarians (H.J.C. or E.C.W.) in MEDLINE, Embase, and CINAHL Complete on March 10, 2021, and run again on May 12, 2022, for all human studies including children 18 years and younger. There were no language or date limitations. Only randomized clinical trials were included in the review. Further details of the literature search are provided in the eMethods in Supplement 1, and the complete search strategy is provided in eTable 2 in Supplement 1. Data abstraction was done by a pair of independent reviewers using a standardized data collection form in REDCap,^9^ and discrepancies between the 2 reviewers were resolved by a third reviewer. Risk of bias of included studies was assessed using the Cochrane tool for the assessment of risk of bias in randomized trials (RoB version 2.0).^10^

### Statistical Analysis

HFNC, CPAP, and BiPAP were the experimental nodes (interventions in a network plot), and COT was considered the reference node in the network meta-analysis. We performed the analysis using a bayesian analytic framework. A bayesian approach has been preferred for network meta-analyses since it is better able to handle studies with very few events and produce probability and ranking outputs that are intuitive to end users.^11^ A bayesian random-effects model for network meta-analysis was adopted because it assumes and accounts for unexplained heterogeneity across studies (eMethods in Supplement 1).

Different interventions were ranked using the rank probabilities generated by the bayesian approach. We also used the surface under the cumulative ranking curve (SUCRA) to describe the relative ranking of interventions. SUCRA is expressed as a fraction and provides the relative probability of an intervention being the best among all options.^12^ SUCRA of 1 for an intervention indicates that the intervention is certain to be the best among all the interventions tested, while a SUCRA of 0 indicates that the intervention is certain to be the worst among the treatments tested. It is recommended that the ranks be interpreted in the context of the certainty of evidence and the absolute risk reduction of the pairwise comparisons.^13,14^

Using the bayesian framework, we performed a meta-regression analysis to explore the association of age with the effectiveness of NRS on reducing EF (reintubation) and TF. In our model, we assumed a common study-level covariate effect vs the baseline treatment (COT).^15^ We chose to divide studies into 2 groups, those with a mean age 6 months and younger and those with a mean age older than 6 months based on epidemiologic data suggesting higher rates of EF in younger children.^16^ Model comparisons were based on comparing model fit in addition to the deviance information criterion (DIC). DIC is the combination of the penalty incurred for complexity of a model and the deviance for a model.^17^ Models with smaller DIC are preferred to models with larger DIC, and a difference in DIC greater than 7 is considered substantial.^17^

For outcomes with only 2 interventions, we performed standard pairwise meta-analysis with a random-effects model using RevMan version 5.4 (Cochrane Collaboration). The network meta-analysis was conducted using the GeMTC package of R version 3.5.3 (RStudio), and the network plots were created using the multinma package in R version 4.2.2 (R Foundation).^18^ We assessed certainty of evidence using recently published guidance by the GRADE working group (eMethods in Supplement 1).^19,20^

## Results

A total of 11 615 records were screened, 11 441 of which were excluded after reviewing the abstracts. Full texts of the remaining 174 records were reviewed for eligibility. A total of 9 randomized clinical trials fulfilled the eligibility criteria and were included in the analysis. eFigure 1 in Supplement 1 shows the reasons for exclusion of records during the full text review.

The 9 included studies had a total sample size of 1421 participants.^21,22,23,24,25,26,27,28,29^ Characteristics of the studies included in this review along with the details of NRS equipment and the interfaces used are provided in eTable 3 in Supplement 1. Five studies compared COT with NRS; 3 compared COT with HFNC,^21,26,27^ 1 compared COT with CPAP,^25^ and 1 compared COT with BiPAP.^22^ Two trials compared HFNC and CPAP,^23,24^ 1 compared HFNC and BiPAP,^28^ and 1 compared CPAP and BiPAP.^29^ NRS (HFNC, CPAP, and BiPAP) was initiated immediately after extubation (planned NRS) in 6 studies,^21,22,26,27,28,29^ while 1 study used NRS only with the onset of respiratory distress (rescue NRS).^25^ Two studies allowed both planned and rescue NRS.^23,24^ The primary outcome varied between the studies, but all studies reported EF (defined as reintubation within 48 to 72 hours).

The risk of bias profiles for EF is shown in eFigure 2 in Supplement 1. None of the trials were blinded because of the impracticality of blinding NRS. Concealment of the allocation sequence was poorly reported. In the context of lack of blinding, 2 studies were considered to have high risk of bias because they allowed crossover of COT to the other arm. Details of the risk of bias profiles for other outcomes is provided in supplemental figures (eFigures 3-11 in Supplement 1).

### Estimates of Interventions

**Figure 1.**
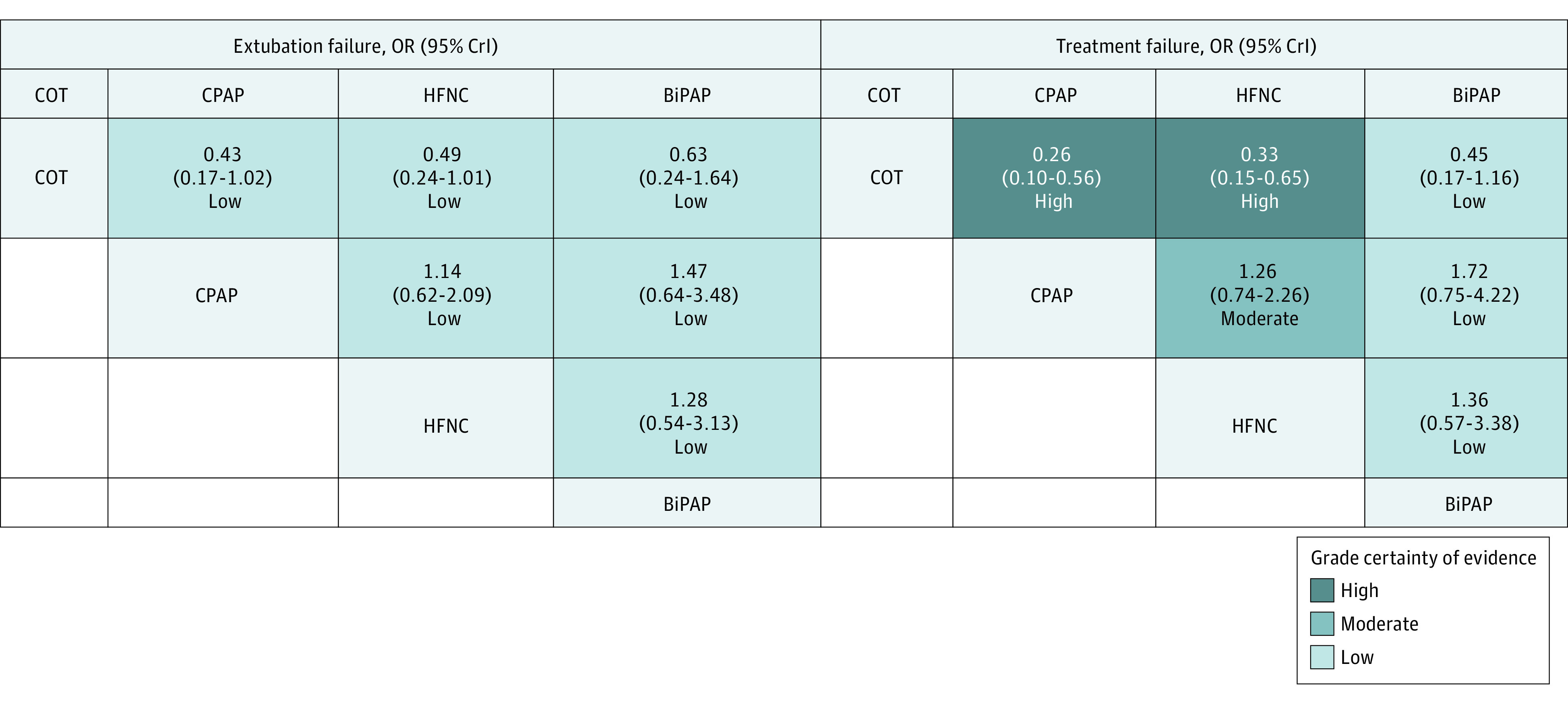
Effect Estimates and Grading of Recommendations Assessment, Development, and Evaluation Certainty of Evidence Rating for Reintubation and Treatment Failure Odds ratios (ORs) and 95% credible intervals (CrIs) are presented. Comparisons between treatments should be read from left to right. ORs less than 1 favor the column-defining treatment for the network estimates. BiPAP indicates bilevel positive airway pressure; COT, conventional oxygen therapy; CPAP, continuous positive airway pressure; HFNC, high-flow nasal cannula.

**Figure 2.**
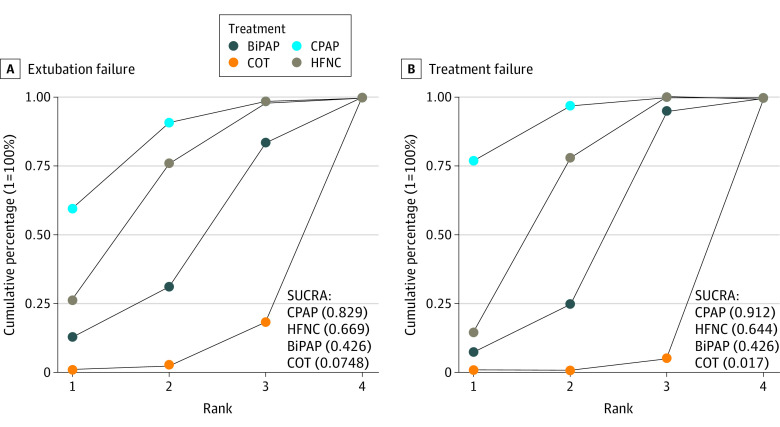
Cumulative Ranks and Surface Under the Cumulative Rank Curve (SUCRA) for Extubation Failure and Treatment Failure Cumulative probability curves and SUCRA values for different noninvasive respiratory support modes. For each mode, the cumulative probability of being ranked first through fourth is displayed. The more the curve for a certain regimen is located toward the upper left corner, the higher its SUCRA value and the better its effectiveness. BiPAP indicates bilevel positive airway pressure; COT, conventional oxygen therapy; CPAP, continuous positive airway pressure; HFNC, high-flow nasal cannula.

**Figure 3.**
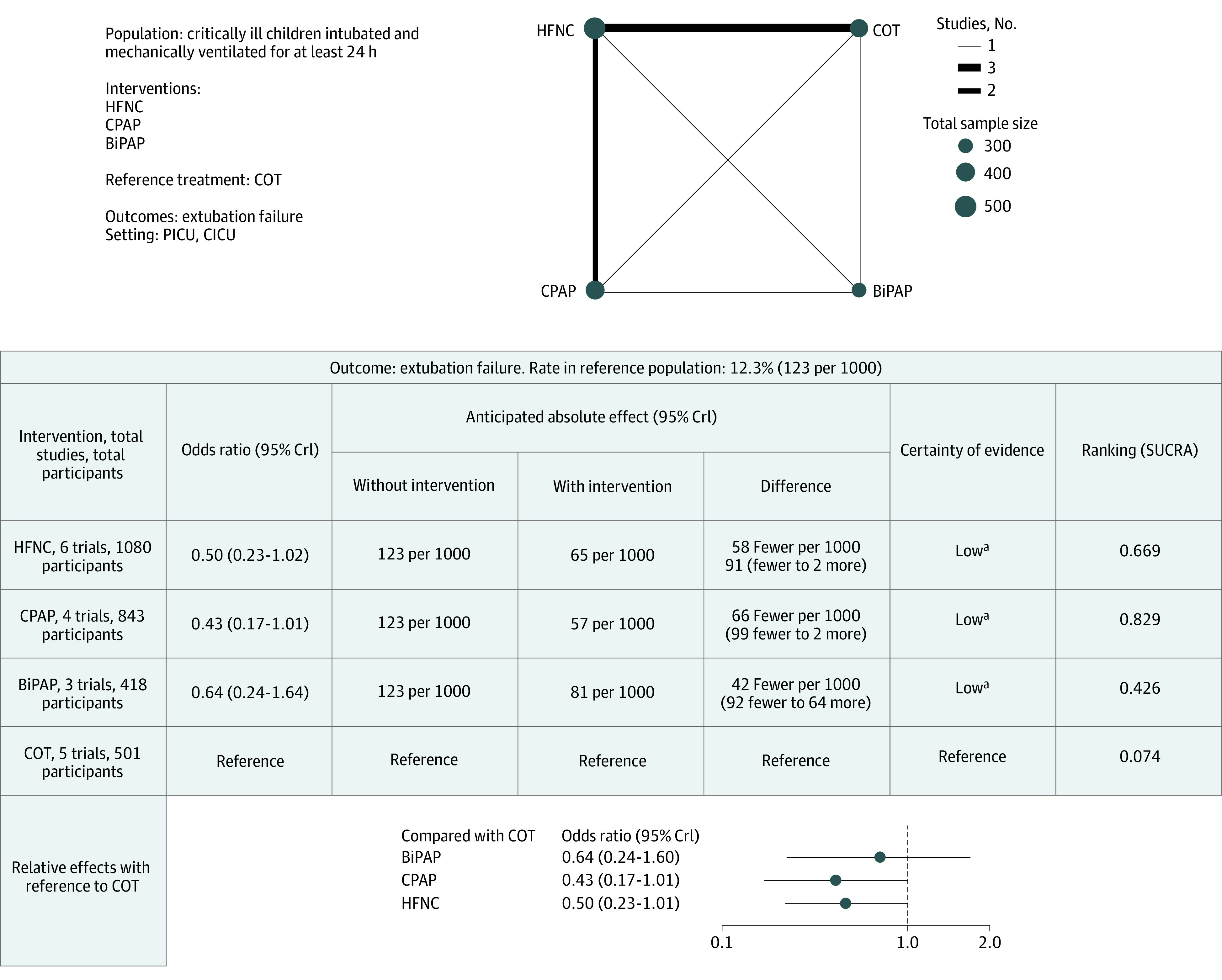
Summary of Findings for Extubation Failure BiPAP indicates bilevel positive airway pressure; COT, conventional oxygen therapy; CPAP, continuous positive airway pressure; CrI, credible interval; HFNC, high-flow nasal cannula. ^a^Downgraded due to serious risk of bias and imprecision.

**Figure 4.**
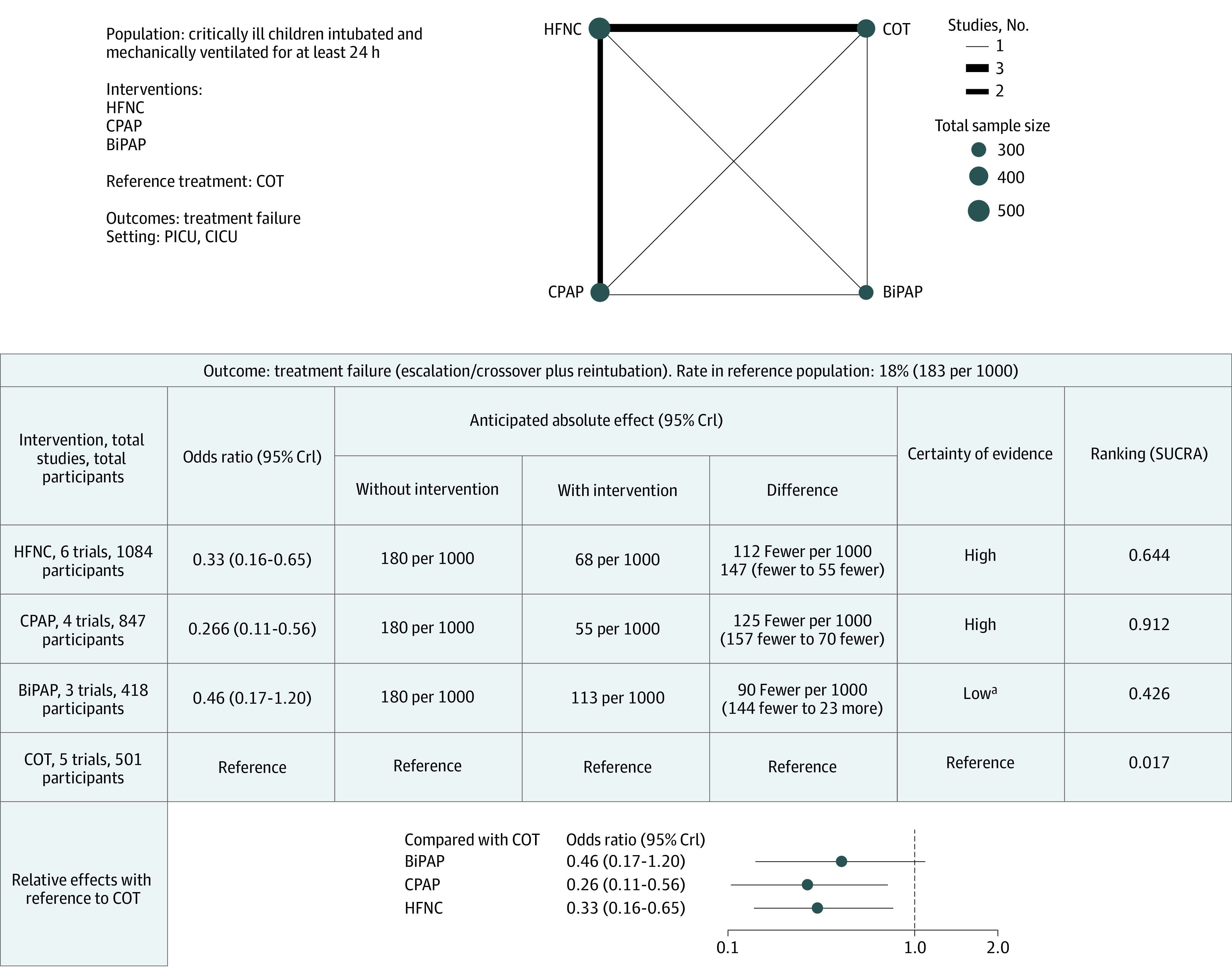
Summary of Findings for Treatment Failure BiPAP indicates bilevel positive airway pressure; CICU, cardiac intensive care unit; COT, conventional oxygen therapy; CPAP, continuous positive airway pressure; CrI, credible interval; HFNC, high-flow nasal cannula. ^a^Downgraded due to serious risk of bias and imprecision.

In the network meta-analysis, all 9 trials reported outcomes for EF and TF. Figure 1 describes the relative effect estimates and absolute estimates reported for EF and TF of COT, HFNC, CPAP, and BiPAP. HFNC, CPAP, and BiPAP were associated with lower rates of EF compared to COT. The largest absolute risk reduction (6%), with a baseline risk of EF of 12%, was seen with CPAP (number needed to treat = 17 per 1000 patients). CPAP had the highest probability of being the best intervention with a SUCRA of 0.83. HFNC, CPAP, and BiPAP appeared to be even more effective with the outcome of reducing TF. Compared to COT, both HFNC (11% reduction) and CPAP (12% reduction) were associated with large absolute reductions in TF with the baseline TF rate of 18%. Like for EF, CPAP had the highest probability of being the best intervention to prevent TF with a SUCRA of 0.91. HFNC was the second ranked intervention and BiPAP the third ranked intervention for both EF and TF. The cumulative ranking curves^30^ for EF and TF are shown in Figure 2. The summary absolute effect sizes of all the comparisons along with the GRADE certainty of evidence estimates is provided in Figure 3 (EF) and Figure 4 (TF).

**Figure 5.**
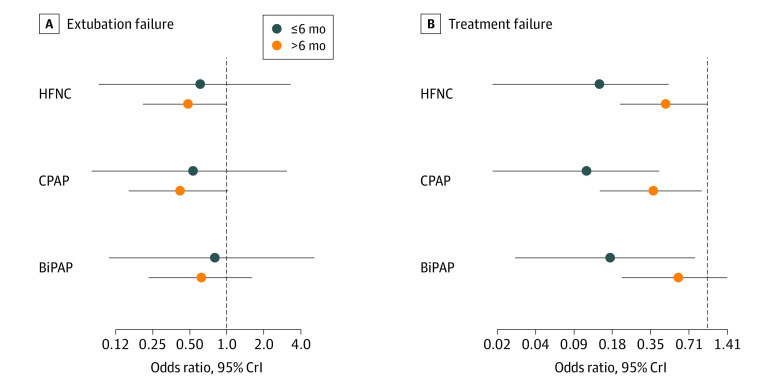
Meta-Regression Analysis for Extubation Failure and Treatment Failure Using Age as an Effect Modifier Forest plot of effect estimates and 95% credible intervals (CrIs) derived from the meta-regression network meta-analysis exploring the impact of age (≤6 months vs >6 months) on the effectiveness of noninvasive respiratory support for preventing extubation failure and treatment failure. BiPAP indicates bilevel positive airway pressure; CICU, cardiac intensive care unit; CPAP, continuous positive airway pressure; HFNC, high-flow nasal cannula.

Age-adjusted subgroup forest plots derived using a meta-regression analysis for EF and TF are shown in Figure 5. The effect estimates appear similar for EF, whereas for TF, NRS appeared to be more effective in infants 6 months and younger. The interaction coefficient B (log odds ratio [OR] with 95% CrI) for EF was 0.25 (−1.60 to 2.06) with a DIC of 31.7. The interaction coefficient for TF was −1.21 (−2.91 to 0.25) with a DIC of 31.4. Thus, the covariate-adjusted models did not offer notable improvement in DIC compared with the unadjusted models (DIC = 30.4), and the 95% CrI of the interaction coefficient includes the possibility of no interaction for both the outcomes. The 95% CrI of the interaction coefficient for TF was close to the level of significance, suggesting that all 3 interventions (HFNC, CPAP, and BiPAP) may be more effective than COT in infants. In this age-adjusted model, CPAP remained the best ranked treatment, with a SUCRA of 0.82 for EF and 0.89 for TF.

A detailed summary of other findings can be found in eTable 4 in Supplement 1 and risk of bias profiles are in eFigures 3-8 in Supplement 1). Compared to COT, hospital length of stay was shorter for HFNC (−8.7 days; 95% CrI, −19.0 to 1.1) and CPAP (−9 days; 95% CrI, −20.0 to 2.4) (eTable 4 in Supplement 1), and the estimates were similar to COT for HFNC (0.03 days; 95% CrI, −1.6 to 1.7) and CPAP (−0.3 days; 95% CrI, −3.2 to 2.6) for PICU length of stay (eTable 5 in Supplement 1).

PICU mortality was reported in 4 trials.^23,24,27,28^ One trial^28^ had 0 events for the HFNC arm, and this study was not included in the network meta-analysis, as there is no standard methodology in bayesian network analyses for dealing with studies with 0 events.^31,32^ COT (3.9% mortality), CPAP (1.2% mortality), and HFNC (2.2% mortality in all studies and 2.5% mortality in studies included in the analysis) had similar rates of PICU mortality (eTable 6 in Supplement 1).

Two trials^26,27^ comparing HFNC and COT had 0 events related to nasal injury, and these were excluded from the analysis. Incidence of nasal injury was modestly elevated for CPAP (3.8%) and HFNC (1.3%) and moderately elevated for BiPAP (8.7%) compared to COT. Compared to HFNC, CPAP (OR, 2.7; 95% CrI, 0.84-13) and BiPAP (OR, 3.1; 95% CrI, 0.80-20) had a nonsignificant trend for increased incidence of nasal injury (eTable 7 in Supplement 1).

COT had 0 events of abdominal distension in 2 trials. The mean incidence of abdominal distension was similar for all NRS modes but modestly higher than COT (HFNC, 2.4%; CPAP, 2.8%; and BiPAP, 3.2%) with no difference between the NRS modes in the network meta-analysis (eTable 8 in Supplement 1). Analysis for all the outcomes reached convergence and none of network loops showed inconsistency.

Three outcomes—hospital mortality, aspiration, and sedation use—were only reported in 2 studies comparing CPAP and HFNC (eTable 9 and eFigures 9-11 in Supplement 1).^23,24^ In a pairwise analysis, hospital mortality was lower with CPAP compared to HFNC with an OR of 0.38 (95% CI, 0.15-0.97). This difference in mortality was largely due to unexplained difference in 1 study where most deaths in the HFNC group (ie, 8 of 13) happened after PICU discharge.^24^ The rates of aspiration (OR, 1.00; 95% CI, 0.21-4.73) and sedation use (OR, 0.95; 95% CI, 0.83-1.09) were not different between the HFNC and CPAP groups (eFigure 12 in Supplement 1). Length of invasive mechanical ventilation prior to extubation was not reported in all the studies, and we could not analyze its impact on NRS efficacy.

## Discussion

There is increasing recognition of potential harms with prolonged use of invasive mechanical ventilation in children.^33,34^ Early liberation from invasive mechanical ventilation, often with the use of postextubation NRS, has been attempted with the aim of reducing the duration of invasive mechanical ventilation without increasing the rates of EF. Many modes of NRS have been studied in children but the optimal mode for postextubation respiratory support remains uncertain.^35^ Using a network meta-analysis model, our study results suggest that HFNC, CPAP, and BiPAP appeared to be better than COT in preventing EF and TF in the 9 included trials. CPAP was likely the best modality for preventing EF and TF. HFNC was likely the second best modality for preventing EF and TF, with an effectiveness only modestly lower than that of CPAP.

The results of the meta-regression analysis did not show a statistically significant interaction with age and should be considered exploratory. Nevertheless, our results suggest a trend of improved efficacy of NRS in children 6 months and younger compared to those older than 6 months. CPAP remained the best NRS mode in infants 6 months and younger.

We used TF as a composite outcome (escalation or crossover of respiratory support plus EF) to describe the real-world practice in which escalation or change to other forms of NRS are often tried before reintubation. Trials that allowed escalation reflect a practice that is closer to real-world postextubation care but obscure the true difference in EF rates between the trial arms. In this systematic review and network meta-analysis, we found a large reduction in TF both with CPAP (12.5% less) and HFNC (11.2% less) compared to COT.

Our report illustrates the trade-offs involved when choosing a NRS modality for postextubation support. Compared to COT, CPAP and HFNC showed large reductions in EF (approximately 6% reduction) and TF (approximately 12% reduction) and possibly hospital length of stay (approximately 9 days reduction). On the other hand, CPAP and BiPAP were associated with high rates of nasal trauma compared to COT (3% to 8% increase) and HFNC (approximately 1% increase). HFNC, CPAP, and BiPAP also had an approximate 2% increase in the incidence of abdominal distension compared to COT. Comparing CPAP and HFNC, both modalities had similar reported effectiveness in preventing EF and TF, although CPAP was ranked higher for both the outcomes. PICU and hospital length of stay, aspiration risk, and sedation use were similar between CPAP and HFNC. CPAP had reduced hospital mortality compared to HFNC, although most of the difference in mortality was after discharge from the PICU, and the cause of the difference is unclear. As most patients, families, and clinicians are likely to value preventing EF over the potential adverse outcomes (eg, pressure injury and abdominal distension), CPAP and HFNC would typically be preferable to COT for postextubation support, especially in children at high risk of EF. EF rates vary across regions and health care settings^36,37^; the absolute risk reduction in EF associated with NRS use will likely increase in settings where the baseline EF rate is higher and where NRS modes can be effectively implemented. A recent network meta-analysis including adult trials studying the efficacy of postextubation NRS suggested increased effectiveness with NRS in patients at higher risk of EF.^38^ We could not perform a similar analysis due to a lack of sufficient number of randomized clinical trials among children at high risk of EF.

### Limitations

There are several limitations to our study. The risk of bias associated with studies resulted in low to very low certainty of evidence in most comparisons and therefore the study results should be interpreted with caution. The generalizability of our analysis is affected by the characteristics of the population included in the trials. Only 2 studies had a mean age older than 1 year, and the mean age in these was younger than 48 months, which might limit generalization of these results to older patients. None of the studies with CPAP or BiPAP had a mean or median age older than 1 year. Further, patients with certain noncardiac congenital abnormalities (eg, congenital diaphragmatic hernia and facial abnormalities) and neurologic or neuromuscular impairment were excluded from 7 and 5 studies, respectively. Thus, the results of this analysis may only be applicable to younger children without such abnormalities. Similarly, CPAP and BiPAP interfaces were varied and included nonocclusive nasal cannulas, which are likely less effective in providing predictable pressures compared to leak-free interfaces. When choosing a NRS mode, considerations of equipment availability, associated costs to patients and the health care system, and the need for a high level of nursing care are also important. These factors vary across health systems and geographic regions and are likely to have an impact on the relative efficacy of different NRS modes. Most trials did not include outcomes or data related to resource utilization or cost. Costs of different NRS modes are not standard across countries and health systems; sometimes overall costs associated with a specific NRS mode become the decisive issue in the choice of NRS to be used in an institution.

## Conclusion

Despite its limitations, this systematic review and network meta-analysis provides evidence of better reported effectiveness with CPAP, HFNC, and BiPAP compared to COT in preventing EF and TF with modest increases in complications such as abdominal distension and nasal injury. CPAP was likely to be the best intervention to prevent EF and TF. Future studies are needed in children older than 2 years and in specific populations at higher risk of EF.

## Notes

Supplement 1. eMethods. Details of Bayesian network analysis and Assessment of certainty of evidence eTable 1. PRISMA NMA Checklist of Items to Include When Reporting A Systematic Review Involving a Network Meta-analysis eTable 2. Search strategies for MEDLINE, Embase, and CINAHL eTable 3. Characteristics of included studies. eTable 4. Summary of findings: Hospital length of stay eTable 5. Summary of findings: PICU length of stay eTable 6. Summary of findings: PICU Mortality eTable 7. Summary of findings: Nasal Injury eTable 8. Summary of findings: Abdominal distension eTable 9. Summary of findings for pair-wise analysis between CPAP and HFNC eFigure 1. PRISMA Flow of information eFigure 2. Risk of bias. Extubation failure eFigure 3. Risk of bias: Treatment failure eFigure 4. Risk of bias: Abdominal distension eFigure 5. Risk of bias: Hospital length of stay eFigure 6. Risk of bias: PICU mortality eFigure 7. Risk of bias: Nasal injury eFigure 8. Risk of bias: PICU LOS eFigure 9. Risk of bias: Hospital mortality eFigure 10. Risk of bias: Sedation use eFigure 11. Risk of bias: Aspiration eFigure 12. Forest plot of effect estimates and 95% confidence intervals of the pairwise metanalysis comparing CPAP and HFNC.

Supplement 2. Data sharing statement
