# Supplementary material for: Association of Extubation Failure Rates With High-Flow Nasal Cannula, Continuous Positive Airway Pressure, and Bilevel Positive Airway Pressure vs Conventional Oxygen Therapy in Infants and Young Children: A Systematic Review and Network Meta-Analysis
Source: JAMA Pediatr. 2023 Jun 5;177(8):774–81. doi: 10.1001/jamapediatrics.2023.1478 (PMC10242512; doi:10.1001/jamapediatrics.2023.1478)
Supplement: Supplement 1. — eMethods. Details of Bayesian network analysis and Assessment of certainty of evidence eTable 1. PRISMA NMA Checklist of Items to Include When Reporting A Systematic Review Involving a Network Meta-analysis eTable 2. Search strategies for MEDLINE, Embase, and CINAHL eTable 3. Characteristics of included studies. eTable 4. Summary of findings: Hospital length of stay eTable 5. Summary of findings: PICU length of stay eTable 6. Summary of findings: PICU Mortality eTable 7. Summary of findings: Nasal Injury eTable 8. Summary of findings: Abdominal distension eTable 9. Summary of findings for pair-wise analysis between CPAP and HFNC eFigure 1. PRISMA Flow of information eFigure 2. Risk of bias. Extubation failure eFigure 3. Risk of bias: Treatment failure eFigure 4. Risk of bias: Abdominal distension eFigure 5. Risk of bias: Hospital length of stay eFigure 6. Risk of bias: PICU mortality eFigure 7. Risk of bias: Nasal injury eFigure 8. Risk of bias: PICU LOS eFigure 9. Risk of bias: Hospital mortality eFigure 10. Risk of bias: Sedation use eFigure 11. Risk of bias: Aspiration eFigure 12. Forest plot of effect estimates and 95% confidence intervals of the pairwise metanalysis comparing CPAP and HFNC. [file jamapediatr-e231478-s001.pdf]

Supplemental Online Content

Iyer NP, Rotta AT, Essouri S, et al. Association of extubation failure rates with high-flow nasal cannula, continuous positive airway pressure, and bilevel positive airway pressure vs conventional oxygen therapy in infants and young children: a systematic review and network meta-analysis. *JAMA Pediatr*. Published online June 5, 2023.
doi:10.1001/jamapediatrics.2023.1478

eMethods. Details of Bayesian network analysis and Assessment of certainty of evidence

eTable 1. PRISMA NMA Checklist of Items to Include When Reporting A Systematic Review Involving a Network Meta-analysis

eTable 2. Search strategies for MEDLINE, Embase, and CINAHL

eTable 3. Characteristics of included studies.

eTable 4. Summary of findings: Hospital length of stay

eTable 5. Summary of findings: PICU length of stay

eTable 6. Summary of findings: PICU Mortality

eTable 7. Summary of findings: Nasal Injury

eTable 8. Summary of findings: Abdominal distension

eTable 9. Summary of findings for pair-wise analysis between CPAP and HFNC

eFigure 1. PRISMA Flow of information

eFigure 2. Risk of bias. Extubation failure

eFigure 3. Risk of bias: Treatment failure

eFigure 4. Risk of bias: Abdominal distension

eFigure 5. Risk of bias: Hospital length of stay

eFigure 6. Risk of bias: PICU mortality

eFigure 7. Risk of bias: Nasal injury

eFigure 8. Risk of bias: PICU LOS

© 2023 Iyer NP et al. *JAMA Pediatrics.*

eFigure 9. Risk of bias: Hospital mortality

eFigure 10. Risk of bias: Sedation use

eFigure 11. Risk of bias: Aspiration

eFigure 12. Forest plot of effect estimates and 95% confidence intervals of the pairwise metanalysis comparing CPAP and HFNC.

This supplemental material has been provided by the authors to give readers additional information about their work.

© 2023 Iyer NP et al. *JAMA Pediatrics.*

eMethods

Literature search

Pairs of reviewers independently screened the title and abstracts and performed full text review. Any conflicts were resolved by a third reviewer. Title, abstract screening and full text review were performed using the systematic review software, Covidence (Covidence systematic review software, Veritas Health Innovation, Melbourne, Vic, Australia. (www.covidence.org). We used the following eligibility criteria:

a) Patients: We included studies conducted in the PICU or the pediatric cardiac intensive care unit (CICU) that were performed on critically ill children up to age 18 years, receiving IMV for more than 24 hours who underwent or were scheduled for planned ventilator liberation. We excluded studies that included preterm infants (37 weeks or less gestation at birth) and where data for older infants and children were not included. We also excluded studies where extubation occurred in the neonatal intensive care unit (NICU) or outside the intensive care units (e.g. operating rooms).

b) Study type: We included randomized trials evaluating the use of post-extubation NRS. We aimed to include all NRS modes using any patient interface.

Details of Bayesian model

For both the main analysis and the meta-regression analysis, we used Bayesian methods with informative priors for the between-trials heterogeneity. An empirical study conducted by Turner et al. provides the basis for choosing a plausible prior for the between-studies variance parameter [in our analysis a log normal distribution (-2.89, 1.91)], which is assumed to be equal across comparisons.[1]

© 2023 Iyer NP et al. *JAMA Pediatrics.*

The analysis was conducted with the Markov Chain Monte Carlo methods.[2] Four Markov chains, yielding 400 000 iterations (100,000 iterations per chain after an initial burn-in of 10,000 and a thinning of 10) generating the posterior distributions of the model parameters, were carried out. Convergence was checked by using the Brooks-Gelman-Rubin diagnostic.[3] The goodness of fit of the model was assessed with residual deviance.[2] The I^2 statistic was used to assess statistical heterogeneity. Inconsistency was determined by the Bayesian p-value calculated using the node splitting approach.[4] The effect of the intervention for dichotomous outcomes was summarized as odds ratio and 95% credible intervals (CrI); for continuous measures data was summarized as mean difference and 95% CrI.

Assessment of certainty of evidence

We assessed certainty of evidence using recently published guidance by the GRADE working group.[5,6] For this analysis, we used a minimally contextualized approach which only considers if the credible intervals include the null effect.[7] Thresholds for ARR were determined by a survey of authors. The authors considered a difference of >3% (>30 per 1000 ARR) in extubation failure, a difference of >6% (>100 per 1000 ARR) in treatment failure (assuming half of patients with treatment failure get reintubated), a difference in PICU LOS of >24 hours and a difference in length of IMV of >12 hours as clinically significant.

© 2023 Iyer NP et al. *JAMA Pediatrics.*

eTable 1. PRISMA NMA Checklist of Items to Include When Reporting A Systematic Review Involving a Network Meta-analysis

Section/Topic	Item #	Checklist Item	Reported on Page #
**TITLE**			
Title	1	Identify the report as a systematic review *incorporating a network meta-analysis (or related form of meta-analysis).*	**1**
**ABSTRACT**			
Structured summary	2	Provide a structured summary including, as applicable:<br>**Background:** main objectives<br>**Methods:** data sources; study eligibility criteria, participants, and interventions; study appraisal; and *synthesis methods, such as network meta-analysis.*<br>**Results:** number of studies and participants identified; summary estimates with corresponding confidence/credible intervals; *treatment rankings may also be discussed. Authors may choose to summarize pairwise comparisons against a chosen treatment included in their analyses for brevity.*<br>**Discussion/Conclusions:** limitations; conclusions and implications of findings.<br>**Other:** primary source of funding; systematic review registration number with registry name.	5-6
**INTRODUCTION**			
Rationale	3	Describe the rationale for the review in the context of what is already known*, including mention of why a network meta-analysis has been conducted.*	**7**
Objectives	4	Provide an explicit statement of questions being addressed, with reference to participants, interventions, comparisons, outcomes, and study design (PICOS).	7
**METHODS**			
Protocol and registration	5	Indicate whether a review protocol exists and if and where it can be accessed (e.g., Web address); and, if available, provide registration information, including registration number.	7-8

© 2023 Iyer NP et al. *JAMA Pediatrics.*

Eligibility criteria	6	Specify study characteristics (e.g., PICOS, length of follow-up) and report characteristics (e.g., years considered, language, publication status) used as criteria for eligibility, giving rationale. *Clearly describe eligible treatments included in the treatment network, and note whether any have been clustered or merged into the same node (with justification).*	**8-9**
Information sources	7	Describe all information sources (e.g., databases with dates of coverage, contact with study authors to identify additional studies) in the search and date last searched.	9
Search	8	Present full electronic search strategy for at least one database, including any limits used, such that it could be repeated.	eTable2
Study selection	9	State the process for selecting studies (i.e., screening, eligibility, included in systematic review, and, if applicable, included in the meta-analysis).	eMethods
Data collection process	10	Describe method of data extraction from reports (e.g., piloted forms, independently, in duplicate) and any processes for obtaining and confirming data from investigators.	9
Data items	11	List and define all variables for which data were sought (e.g., PICOS, funding sources) and any assumptions and simplifications made.	8-9
**Geometry of the network**	**S1**	Describe methods used to explore the geometry of the treatment network under study and potential biases related to it. This should include how the evidence base has been graphically summarized for presentation, and what characteristics were compiled and used to describe the evidence base to readers.	**Table 2, Table 3**
Risk of bias within individual studies	12	Describe methods used for assessing risk of bias of individual studies (including specification of whether this was done at the study or outcome level), and how this information is to be used in any data synthesis.	9
Summary measures	13	State the principal summary measures (e.g., risk ratio, difference in means). *Also describe the use of additional summary measures assessed, such as treatment rankings and surface under the cumulative ranking curve (SUCRA) values, as well as modified approaches used to present summary findings from meta-analyses.*	9-10
Planned methods of analysis	14	Describe the methods of handling data and combining results of studies for each network meta-	9-10 eMethods

© 2023 Iyer NP et al. *JAMA Pediatrics.*

		analysis. This should include, but not be limited to:<br>• *Handling of multi-arm trials;*<br>• *Selection of variance structure;*<br>• *Selection of prior distributions in Bayesian analyses; and*<br>• *Assessment of model fit.*	
**Assessment of Inconsistency**	**S2**	Describe the statistical methods used to evaluate the agreement of direct and indirect evidence in the treatment network(s) studied. Describe efforts taken to address its presence when found.	eMethods
Risk of bias across studies	15	Specify any assessment of risk of bias that may affect the cumulative evidence (e.g., publication bias, selective reporting within studies).	**9**
Additional analyses	16	Describe methods of additional analyses if done, indicating which were pre-specified. This may include, but not be limited to, the following:<br>• Sensitivity or subgroup analyses;<br>• Meta-regression analyses;<br>• *Alternative formulations of the treatment network; and*<br>• *Use of alternative prior distributions for Bayesian analyses (if applicable).*	**9**
**RESULTS†**			
Study selection	17	Give numbers of studies screened, assessed for eligibility, and included in the review, with reasons for exclusions at each stage, ideally with a flow diagram.	11-12
**Presentation of network structure**	**S3**	Provide a network graph of the included studies to enable visualization of the geometry of the treatment network.	**Table 2, 3**
**Summary of network geometry**	**S4**	Provide a brief overview of characteristics of the treatment network. This may include commentary on the abundance of trials and randomized patients for the different interventions and pairwise comparisons in the network, gaps of evidence in the treatment network, and potential biases reflected by the network structure.	**Table 2,3**
Study characteristics	18	For each study, present characteristics for which data were extracted (e.g., study size, PICOS, follow-up period) and provide the citations.	eTable3

© 2023 Iyer NP et al. *JAMA Pediatrics.*

Risk of bias within studies	19	Present data on risk of bias of each study and, if available, any outcome level assessment.	eFigure 2-11
Results of individual studies	20	For all outcomes considered (benefits or harms), present, for each study: 1) simple summary data for each intervention group, and 2) effect estimates and confidence intervals. *Modified approaches may be needed to deal with information from larger networks.*	**11-14**
Synthesis of results	21	Present results of each meta-analysis done, including confidence/credible intervals. *In larger networks, authors may focus on comparisons versus a particular comparator (e.g. placebo or standard care), with full findings presented in an appendix. League tables and forest plots may be considered to summarize pairwise comparisons.* If additional summary measures were explored (such as treatment rankings), these should also be presented.	**11-14**
**Exploration for inconsistency**	**S5**	Describe results from investigations of inconsistency. This may include such information as measures of model fit to compare consistency and inconsistency models, *P* values from statistical tests, or summary of inconsistency estimates from different parts of the treatment network.	**14**
Risk of bias across studies	22	Present results of any assessment of risk of bias across studies for the evidence base being studied.	eFigure 2-11 and Summary of findings table
Results of additional analyses	23	Give results of additional analyses, if done (e.g., sensitivity or subgroup analyses, meta-regression analyses*, alternative network geometries studied, alternative choice of prior distributions for Bayesian analyses,* and so forth).	**12-13**
**DISCUSSION**			
Summary of evidence	24	Summarize the main findings, including the strength of evidence for each main outcome; consider their relevance to key groups (e.g., healthcare providers, users, and policy-makers).	14-15
Limitations	25	Discuss limitations at study and outcome level (e.g., risk of bias), and at review level (e.g., incomplete retrieval of identified research, reporting bias). *Comment on the validity of the assumptions, such as transitivity and consistency. Comment on any concerns regarding network geometry (e.g., avoidance of certain comparisons).*	16-17

© 2023 Iyer NP et al. *JAMA Pediatrics.*

Conclusions	26	Provide a general interpretation of the results in the context of other evidence, and implications for future research.	17
**FUNDING**			2-3
Funding	27	Describe sources of funding for the systematic review and other support (e.g., supply of data); role of funders for the systematic review. This should also include information regarding whether funding has been received from manufacturers of treatments in the network and/or whether some of the authors are content experts with professional conflicts of interest that could affect use of treatments in the network.	

PICOS = population, intervention, comparators, outcomes, study design.
* Text in italics indicateS wording specific to reporting of network meta-analyses that has been added to guidance from the PRISMA statement.
† Authors may wish to plan for use of appendices to present all relevant information in full detail for items in this section.

© 2023 Iyer NP et al. *JAMA Pediatrics.*

eTable 2. Search strategies for MEDLINE, Embase, and CINAHL

MEDLINE (Ovid)

Databases selected: Ovid MEDLINE(R) and Epub Ahead of Print, In-Process, In-Data-Review & Other Non-Indexed Citations, Daily and Versions(R)

Line	Query
1	Continuous Positive Airway Pressure/
2	Continuous Positive Airway Pressure*.mp.
3	CPAP.mp.
4	1 or 2 or 3
5	exp Sleep Apnea Syndromes/
6	sleep apnea*.mp.
7	5 or 6
8	4 not 7
9	(extubation* adj2 (readiness or failure* or outcome*)).mp.
10	((face or nasal) adj mask ventilat*).mp.
11	helmet ventilat*.mp.
12	((High-flow or highflow) adj3 nasal cannula*).mp.
13	((high-flow or highflow or humidified) adj3 oxygen*).mp.
14	(negative pressure adj2 ventilator*).mp.
15	NIV.mp.
16	Noninvasive Ventilation/
17	Noninvasive Ventilation*.mp.
18	Non invasive Ventilation*.mp.
19	Oxygen Inhalation Therapy/
20	Oxygen inhalat* therap*.mp.
21	8 or 9 or 10 or 11 or 12 or 13 or 14 or 15 or 16 or 17 or 18 or 19 or 20
22	Adolescent/
23	Adolescen*.mp.
24	Teen*.mp.
25	Youth*.mp.
26	exp Child/
27	Child*.mp.
28	Infant/
29	Infant, Newborn/
30	Infant*.mp.
31	Infanc*.mp.
32	Newborn*.mp.
33	Neonat*.mp.
34	Pediatrics/
35	P?ediatric*.mp.
36	Hospitals, Pediatric/

© 2023 Iyer NP et al. *JAMA Pediatrics.*

37	Intensive Care Units, Pediatric/
38	PICU*.mp.
39	(Kid or kids).mp.
40	Toddler*.mp.
41	22 or 23 or 24 or 25 or 26 or 27 or 28 or 29 or 30 or 31 or 32 or 33 or 34 or 35 or 36 or 37 or 38 or 39 or 40
42	(Adaptive adj2 Support Ventilat*).mp.
43	Airway Extubation/
44	Airway extubat*.mp.
45	Artificial Respirati*.mp.
46	((intubation or extubation*) adj3 (airway or tracheal or intratracheal or endotracheal)).mp.
47	exp Intermittent Positive-Pressure Breathing/
48	Intermittent Positive-Pressure Breathing.mp.
49	exp Intermittent Positive-Pressure Ventilation/
50	Intermittent Positive-Pressure Ventilat*.mp.
51	Intubation, Intratracheal/
52	Mechanical Ventilat*.mp.
53	Neurally Adjusted Ventilatory Assist*.mp.
54	open lung ventilat*.mp.
55	Peep.mp.
56	Positive End Expiratory Pressure*.mp.
57	exp Positive-Pressure Respiration/
58	Positive-Pressure Ventilat*.mp.
59	pressure controlled ventilat*.mp.
60	Proportional Assist Ventilat*.mp.
61	Reintubat*.mp.
62	Respiration, Artificial/
63	Respirator Weaning*.mp.
64	Ventilator*.mp.
65	(Ventilat* adj3 Liberation*).mp.
66	exp Ventilators, Mechanical/
67	exp Ventilator Weaning/
68	Ventilator* Weaning*.mp.
69	Ventilation Weaning*.mp.
70	42 or 43 or 44 or 45 or 46 or 47 or 48 or 49 or 50 or 51 or 52 or 53 or 54 or 55 or 56 or 57 or 58 or 59 or 60 or 61 or 62 or 63 or 64 or 65 or 66 or 67 or 68 or 69
71	21 and 41 and 70

Embase (Elsevier)

Line	Query

© 2023 Iyer NP et al. *JAMA Pediatrics.*

#84	#22 AND #44 AND #83
#83	#45 OR #46 OR #47 OR #48 OR #49 OR #50 OR #51 OR #52 OR #53 OR #54 OR #55 OR #56 OR #57 OR #58 OR #59 OR #60 OR #61 OR #62 OR #63 OR #64 OR #65 OR #66 OR #67 OR #68 OR #69 OR #70 OR #71 OR #72 OR #73 OR #74 OR #75 OR #76 OR #77 OR #78 OR #79 OR #80 OR #81 OR #82
#82	'artificial respirati*'
#81	'volume controlled ventilation'/exp
#80	'ventilation weaning*'
#79	'ventilator* weaning*'
#78	'ventilator weaning'/de
#77	'mechanical ventilator'/de
#76	ventilat* NEAR/3 liberation*
#75	ventilator*
#74	'ventilator'/de
#73	'tracheal extubation'/de
#72	'respirator weaning*'
#71	'artificial ventilation'/de
#70	reintubat*
#69	'protective ventilation'/exp
#68	'proportional assist ventilat*'
#67	'pressure support ventilation'/de
#66	'pressure controlled ventilat*'
#65	'pressure controlled ventilation'/de
#64	'positive-pressure ventilat*'
#63	'positive pressure ventilation'/de
#62	'positive end expiratory pressure*'
#61	'positive end expiratory pressure ventilation'/exp
#60	peep

© 2023 Iyer NP et al. *JAMA Pediatrics.*

#59	'open lung ventilat*'
#58	'noninvasive positive pressure ventilation'/exp
#57	'neurally adjusted ventilatory assist*'
#56	'mechanical ventilat*'
#55	'inverse ratio ventilation'/de
#54	'invasive ventilation'/exp
#53	'endotracheal intubation'/exp
#52	'intermittent positive-pressure ventilat*'
#51	'intermittent positive pressure ventilation'/exp
#50	'intermittent positive-pressure breathing'
#49	'intermittent mandatory ventilation'/exp
#48	(intubation* OR extubation*) NEAR/3 (airway OR tracheal OR intratracheal OR endotracheal)
#47	'airway extubat*'
#46	'extubation'/de
#45	adaptive NEAR/2 support NEXT/1 ventilat*
#44	#23 OR #24 OR #25 OR #26 OR #27 OR #28 OR #29 OR #30 OR #31 OR #32 OR #33 OR #34 OR #35 OR #36 OR #37 OR #38 OR #39 OR #40 OR #41 OR #42 OR #43
#43	toddler*
#42	'toddler'/exp
#41	kid OR kids
#40	picu*
#39	'pediatric intensive care unit'/de
#38	p$ediatric*
#37	'pediatrics'/de
#36	neonat*
#35	newborn*

© 2023 Iyer NP et al. *JAMA Pediatrics.*

#34	infanc*
#33	infant*
#32	'newborn'/exp
#31	'infancy'/exp
#30	'infant'/exp
#29	child*
#28	'child'/exp
#27	youth*
#26	teen*
#25	adolescen*
#24	'adolescence'/de
#23	'adolescent'/exp
#22	#4 OR #5 OR #6 OR #7 OR #8 OR #9 OR #10 OR #11 OR #12 OR #13 OR #14 OR #15 OR #16 OR #21
#21	#19 NOT #20
#20	#17 OR #18
#19	#1 OR #2 OR #3
#18	'sleep apnea*'
#17	'sleep disordered breathing'/exp
#16	'oxygen inhalat* therap*'
#15	'non-invasive ventilat*'
#14	'noninvasive ventilat*'
#13	'noninvasive ventilation'/de
#12	niv
#11	'negative pressure' NEAR/2 ventilat*
#10	('high flow' OR highflow OR humidified) NEAR/3 oxygen*
#9	'high flow oxygen therapy'/de
#8	('high flow' OR highflow OR humidified) NEAR/3 'nasal cannula*'

© 2023 Iyer NP et al. *JAMA Pediatrics.*

#7	'helmet ventilat*'
#6	'heated humidifier'/de
#5	(face OR nasal) NEXT/1 'mask ventilat*'
#4	extubation* NEAR/2 (readiness OR failure* OR outcome*)
#3	cpap
#2	'continuous positive airway pressure*'
#1	'continuous positive airway pressure'/de

CINAHL Complete (EBSCO)

Line	Query
S73	S20 AND S71 AND S72
S72	S70 OR S69 OR S68 OR S67 OR S66 OR S65 OR S64 OR S63 OR S62 OR S61 OR S60 OR S59 OR S58 OR S57 OR S56 OR S55 OR S54 OR S53 OR S52 OR S51 OR S50 OR S49 OR S48 OR S47 OR S46 OR S45 OR S44 OR S43 OR S42 OR S41 OR S40 OR S39
S71	S38 OR S37 OR S36 OR S35 OR S34 OR S33 OR S32 OR S31 OR S30 OR S29 OR S28 OR S27 OR S26 OR S25 OR S24 OR S23 OR S22 OR S21
S70	adaptive N2 support ventilat*
S69	(MH "Extubation")
S68	airway extubat*
S67	artificial respirati*
S66	(intubation* OR extubation*) N3 (airway OR tracheal OR intratracheal OR endotracheal)
S65	(MH "Intermittent Positive Pressure Breathing")
S64	Intermittent Positive-Pressure Breathing
S63	(MH "Intermittent Positive Pressure Ventilation")
S62	Intermittent Positive-Pressure Ventilat*

© 2023 Iyer NP et al. *JAMA Pediatrics.*

S61	(MH "Intubation, Intratracheal")
S60	(MH "Inverse Ratio Ventilation")
S59	(MH "Mandatory Minute Volume Ventilation")
S58	mechanical ventilat*
S57	neurally adjusted ventilatory assist*
S56	open lung ventilat*
S55	peep
S54	(MH "Positive End-Expiratory Pressure")
S53	Positive End Expiratory Pressure*
S52	(MH "Positive Pressure Ventilation")
S51	positive-pressure ventilat*
S50	pressure controlled ventilat*
S49	(MH "Pressure Support Ventilation")
S48	proportional assist ventilat*
S47	reintubat*
S46	(MH "Respiration, Artificial")
S45	'respirator weaning*'
S44	ventilator*
S43	ventilat* N3 liberation*
S42	(MH "Ventilators, Mechanical")
S41	(MH "Ventilator Weaning")
S40	ventilator* weaning*
S39	Ventilation Weaning*
S38	(MH "Adolescence+")

S37	Adolescen*
S36	Teen*
S35	Youth*
S34	(MH "Child") OR (MH "Child, Hospitalized") OR (MH "Child, Medically Fragile") OR (MH "Child, Preschool")
S33	Child*
S32	(MH "Infant") OR (MH "Infant, Hospitalized") OR (MH "Infant, High Risk")
S31	(MH "Infant, Newborn")
S30	Infant*
S29	Infanc*
S28	Newborn*
S27	Neonat*
S26	(MH "Pediatrics")
S25	P#ediatric*
S24	(MH "Intensive Care Units, Pediatric")
S23	PICU*
S22	Kid OR kids
S21	Toddler*
S20	S8 OR S9 OR S10 OR S11 OR S12 OR S13 OR S14 OR S15 OR S16 OR S17 OR S18 OR S19
S19	(MH "Ventilation, Negative Pressure")
S18	"oxygen inhalat* therap*"
S17	"non invasive ventilat*"
S16	"noninvasive ventilat*"
S15	niv

S14	"negative pressure" N2 ventilat*
S13	("high flow" OR highflow OR humidified) N3 oxygen*
S12	("high flow" OR highflow OR humidified) N3 "nasal cannula*"
S11	"helmet ventilat*"
S10	(face OR nasal) N1 "mask ventilat*"
S9	Extubation* N2 (readiness OR failure* OR outcome*)
S8	S6 NOT S7
S7	S4 OR S5
S6	S1 OR S2 OR S3
S5	"sleep apnea*"
S4	(MH "Sleep Apnea Syndromes+")
S3	CPAP
S2	"continuous positive airway pressure*"
S1	(MH "Continuous Positive Airway Pressure")

eTable 3. Characteristics of included studies.

Study	Sample size	Population	Prophylactic or Rescue	Treatment arms
Akyildiz, 2018[8]	100	Pediatric patients intubated for more than 24 hours, redominantly medical subjects. Subjects with cyanotic heart	Prophylactic	HFNC 1-2L/kg to a max 25L/min. COT was delivered by either

© 2023 Iyer NP et al. *JAMA Pediatrics.*

		disease and those who failed spontaneous breathing trial (SBT) excluded. Average age was 27 and 52 months for High Flow Nasal Cannula (HFNC) and Conventional Oxygen Therapy (COT) groups respectively.<br>Patients with diaphragmatic hernia or paralysis, cyanotic congenital heart disease with unrepaired or palliated right to left intracardiac shunt, and presence of a tracheostomy tube were excluded.		nasal cannula or a simple face mask with the same saturation (SpO2) target.
Fioretto, 2015[9]	108	Age 1mo-3 years, passed SBT and high risk of extubation failure [a) Invasive Mechanical ventilation (IMV) for more than 15 days; b) use of inotropic agents for more than 48 hours; c) continuous intravenous administration of sedatives/analgesics d) age between 1 and 3 month; e) mean airway pressure (Paw) greater than 8.5; f) Inspired oxygen (FiO2) greater than 0.4 and oxygenation index (OI) greater than 4.5 immediately before extubation; g) underlying cardiac or pulmonary disease; h) congestive heart failure; i) hypercapnia.<br>Patients with neuromuscular disease, or those with contraindications for NIV (coma or the inability to protect the airway, nonacceptance of NIV by the patient, hemodynamic instability, shock, cardiac arrhythmia, cranial or facial trauma or surgery that could prevent NIV use, abdominal	Prophylactic	Noninvasive ventilation group: EPAP 5 cm H2O and IPAP 15 cmH2O, PS 10 cm H2O. EPAP was titrated up to 10 cm H2O, IPAP was titrated up to 20 cmH2O, and FiO2 was titrated up to 0.6, as needed. Nasal or face mask used.<br>Oxygen therapy: standard nasal catheter to maintain SpO22>92%

© 2023 Iyer NP et al. *JAMA Pediatrics.*

		distention, nausea or vomiting, recent gastric or esophageal surgery, active gastrointestinal hemorrhage, and undrained pneumothorax) were excluded from the study.		
Ramnarayan, 2018[10]	84	Patient's age between > 36 weeks corrected for gestation and < 16 years. Predominantly medical subjects (including patient with different upper and lower respiratory, cardiac, neurological, and neuromuscular disease). Subjects in Rescue group needed to also satisfy one or more of the following criteria a) hypoxia (oxygen saturation < 92% in FiO_2 > 0.40, or equivalent); b) acute respiratory acidosis (pH < 7.3 with a concomitant partial pressure of carbon dioxide (pCO_2) > 6.5 kPa); c) moderate respiratory distress (use of accessory muscles, subcostal and intercostal recession, tachypnoea for age, grunting). ~55% in both groups <1 yr age.<br>Patients with mid-facial/craniofacial anomalies (Unrepaired cleft palate, choanal atresia) or recent craniofacial surgery were excluded from the study.	Prophylactic (62%) and Rescue (38%)	2L/kg/min (<10kg), HFNC rate weight based up to 50L/min in >60kg.<br>CPAP: 6-8cmH2O. No restrictions on type of interface- any of the following could be used: mask, nasal prong, helmet.
Ramnarayan, 2022[11]	553	Patient age from birth (>36 weeks' corrected gestational age) up to 15 years. Median ag 3mo in both groups. Mixed indications for IMV(medical and postoperative). Bronchiolitis overrepresented in CPAP (44.9%) vs HFNC (34.5%) and Cardiac	Prophylactic (63%) and rescue (37%)	2L/kg/min (<12kg), HFNC rate weight based up to 50L/min in >50kg.<br>CPAP: 6-8cmH2O. No restrictions on type of interface- any of

© 2023 Iyer NP et al. *JAMA Pediatrics.*

		indications overrepresented in HFNC (28.8%) vs CPAP (20.2%).<br>Patients with tracheostomy in place, on home non-invasive ventilation prior to admission, midfacial/craniofacial anomalies (unrepaired cleft palate, choanal atresia), or recent craniofacial surgery were excluded.		the following could be used: mask, nasal prong, helmet.
Rodriguez, 2002[12]	25	Predominantly medical subjects. Only those with signs of post-extubation laryngeal edema (using modified Downes and Raphaely's score). 4 and 3.5 mo median ages of COT and CPAP.<br>Patients with a history of anatomical or acquired abnormalities of upper airway were excluded.	Rescue	Control: L-adrenaline every 15-60 minutes, oxygen.<br>CPAP 5-12 cmH2O using nasal cannula in children <2 years age and nasal masks in older children.
Testa, 2014[13]	89	< 18 months, elective cardiac surgery (cyanotic and acyanotic) with CPB and a Risk Adjustment for Congenital Heart Surgery (RACHS) score of 2 and above.<br>Patients with major congenital malformations or neuromuscular disease were excluded.	Prophylactic	HFNC: 2L/kg/min<br>Oxygen therapy: max 2L/min
Wijakprasert, 2018[14]	152	Predominantly medical subjects intubated for at least 24 hours. Pediatric patients between 29 days and 15 years of age, mean age of 42 mo and 32 mo for HFNC and COT groups.<br>Patient with neuromuscular disease, tracheostomy, and nasal abnormalities were excluded.	Prophylactic	HFNC: 1L/kg/min initially, titrated upwards<br>COT: 10L/min
Li Xiaoqing, 2022[15]	102	Predominantly medical causes of IMV for >24 hours- severe	Prophylactic	BiPAP using nasal cannula in children

© 2023 Iyer NP et al. *JAMA Pediatrics.*

		pneumonia, heart failure and sepsis. Ages between 1 month to 14 years old were enrolled and randomly assigned to treatment group 1, Bilevel Positive Airway Pressure (BPAP) (n=55) and treatment group 2, HFNC (n=47). Patient with central respiratory failure and neuromuscular diseases were excluded.		<15 kg and nasal masks in bigger children. HFNC: 1-2L/kg for infants and toddlers, pre-school children. For older children, initial setting of 20L/min.
Zheng, 2022[16]	186	High-risk infants < 6 months of age with stable hemodynamic status after cardiac surgery. High risk factors included pulmonary hypertension, pneumonia, preoperative or postoperative respiratory failure, oxygenation index > 8, PaO2 /FIO2 < 200 mm Hg, and ARDS. After extubation, all infants were intravenously injected with methylprednisolone sodium succinate 1 mg/kg for the prevention of laryngeal edema. Subjects with excessive secretions were given an intravenous infusion of ambroxol hydrochloride to facilitate mucociliary clearance and chest physical therapy. Patients with congenital thoracic and abdominal malformations, those who received postoperative extracorporeal membrane oxygenation support or preoperative tracheotomy and intubation were excluded.	Prophylactic	The initial BiPAP setting 3–6 cm H2O (low) and 8–10 cm H2O (high), rate 20–30/min. Initial nasal CPAP setting was 3–6 cm H2O; the oxygen flow was 6–8 L/min. Both applied using silicone binasal prongs.

© 2023 Iyer NP et al. *JAMA Pediatrics.*

eTable 4. Summary of findings: Hospital length of stay

Effects of estimates and certainty of evidence for post-extubation noninvasive respiratory support

© 2023 Iyer NP et al. *JAMA Pediatrics.*

Population: Critically ill children intubated and mechanically ventilated for at least 24 hours.
Setting: PICU, CICU
Interventions:
High Flow Nasal Cannula (HFNC)
Continuous Positive Airway Pressure (CPAP)
Reference treatment: Conventional Oxygen Therapy (COT)
Outcomes: Hospital length of stay (LOS)

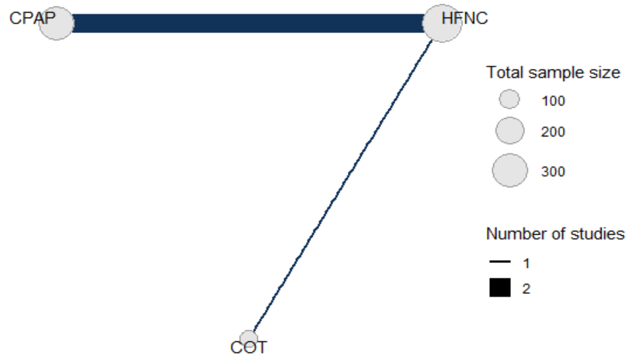

Outcome: Hospital LOS. Hospital LOS in reference population: 26.5 days

**Intervention, Total studies, Total participants**	**Mean difference (95% Credible Interval, CrI)**	**Anticipated absolute effect (95% CrI)**			**Certainty of evidence**	**Ranking (SUCRA)**
		Without intervention	***With intervention***	**Difference**		
HFNC<br>3 trials, 740 participants	-8.7 (-19, 1.1)	26.5 days	17.8 days	8.7 days less (19 days less to 1.1 days more)	Low[a]	0.683
CPAP<br>2 trials, 588 participants	-9.0 (-20, 2.4)	26.5 days	17.5 days	9.0 days less (20 days less to 2.4 days more)	Low[a]	0.708
COT<br>1 trial, 152 participants	Reference	Reference	Reference	Reference	Reference	0.108

Relative effects with reference to COT

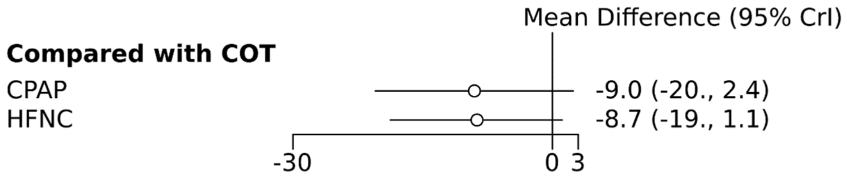

[a]Downgraded due to serious imprecision

Hospital LOS, Effect estimates of all comparisons (mean difference, days):

COT	CPAP	HFNC
COT	-9.06 (-20.8, 2.4)[b]	-8.7 (-19, 1.1)[b]
	CPAP	0.29 (-5.5, 6.0)[c]
		HFNC

[b]Low certainty of effect estimate, [c]Very low certainty of effect estimate

eTable 5. Summary of findings: PICU length of stay

© 2023 Iyer NP et al. *JAMA Pediatrics.*

Effects of estimates and certainty of evidence for post-extubation noninvasive respiratory support

Population: Critically ill children intubated and mechanically ventilated for at least 24 hours.
Setting: PICU, CICU
Interventions:
High Flow Nasal Cannula (HFNC)
Continuous Positive Airway Pressure (CPAP)
Reference treatment: Conventional Oxygen Therapy (COT)
Outcomes: PICU length of stay

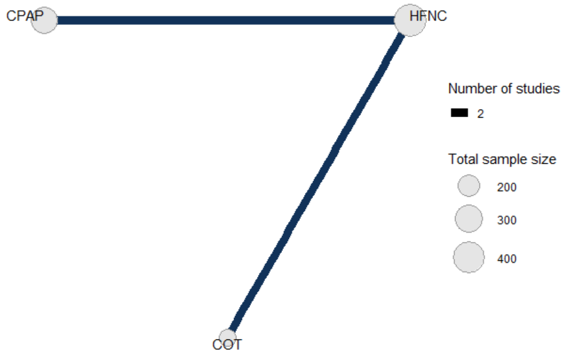

Outcome: PICU LOS. PICU LOS in reference population: 5.85 days

**Intervention, Total studies, Total participants**	**Mean difference (95% Credible Interval, CrI)**	**Anticipated absolute effect (95% CrI)**			**Certainty of evidence**	**Ranking (SUCRA)**
		Without intervention	***With intervention***	**Difference**		
HFNC<br>4 trials, 829 participants	0.04 (-1.6, 1.7)	5.85 days	5.88 days	0.03 days more (1.6 days less to 1.7 days more)	Low	0.494
CPAP<br>2 trials, 588 participants	-0.30 (-3.2, 2.6)	5.85 days	5.35 days	0.3 days less (3.2 days less to 2.6 days more)	Low	0.596
COT<br>2 trials, 241 participants	Reference	Reference	Reference	Reference	Reference	0.409

Relative effects with reference to COT

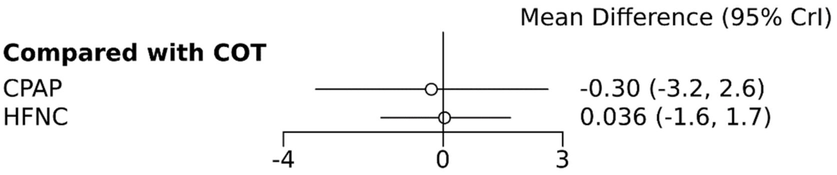

[a]Downgraded due to serious imprecision

PICU LOS, Effect estimates of all comparisons (mean difference, days):

COT	CPAP	HFNC
COT	-0.30 (-3.2, 2.6) [b]	0.04 (-1.5, 1.7) [b]
	CPAP	0.32 (-2.1, 2.7) [b]
		HFNC

[b]Low certainty of effect estimate

eTable 6. Summary of findings: PICU Mortality

© 2023 Iyer NP et al. *JAMA Pediatrics.*

Effects of estimates and certainty of evidence for post-extubation noninvasive respiratory support

Population: Critically ill children intubated and mechanically ventilated for at least 24 hours.
Setting: PICU, CICU
Interventions:
High Flow Nasal Cannula (HFNC)
Continuous Positive Airway Pressure (CPAP)
Bi-Level Positive Airway Pressure (BiPAP)
Reference treatment: Conventional Oxygen Therapy (COT)
Outcomes: PICU Mortality

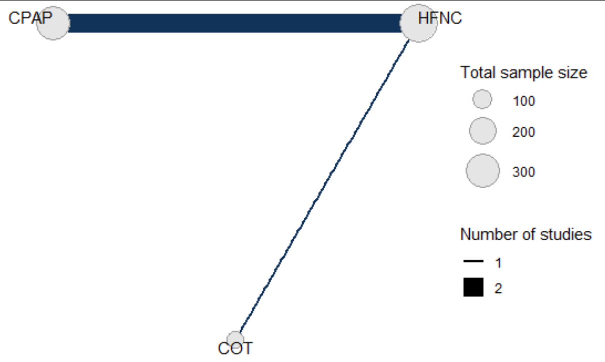

Outcome: PICU Mortality. Rate in reference population: 3.9% (39 per 1000)

Intervention, Total studies, Total participants	Odds ratio (95% Credible Interval, CrI)	Anticipated absolute effect (95% CrI)			Certainty of evidence	Ranking (SUCRA)
		Without intervention	*With intervention*	Difference		
HFNC<br>3 trials with non-zero events, 780 participants	1.3 (0.26, 7.7)	39 per 1000	50 per 1000	11 more per 1000<br>(29 fewer to 199 more)	Very low[a]	0.459
CPAP<br>1 trial, 544 participants	0.96 (0.11, 8.5)	39 per 1000	37 per 1000	2 fewer per 1000<br>(35 fewer to 217 more)	Very low[a]	0.883
COT.<br>1 trial, 152 participants	Reference	Reference	Reference	Reference	Reference	0.618
Relative effects with reference to COT (sensitivity analysis)	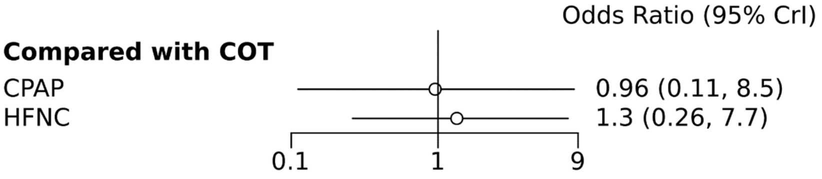					

[a]Downgraded due to serious risk of bias and serious imprecision.

PICU mortality, Effect estimates of all comparisons (Odds ratio, 95% CrI):

COT	CPAP	HFNC
COT	0.96 (0.11, 8.5) [b]	1.34 (0.26, 7.7) [b]
	CPAP	1.38 (0.32, 6.2) [b]
		HFNC

[b]Very low certainty of effect estimates

eTable 7. Summary of findings: Nasal Injury

© 2023 Iyer NP et al. *JAMA Pediatrics.*

Effects of estimates and certainty of evidence for post-extubation noninvasive respiratory support

Population: Critically ill children intubated and mechanically ventilated for at least 24 hours.
Setting: PICU, CICU
Interventions:
Continuous Positive Airway Pressure (CPAP)
Bi-Level Positive Airway Pressure (BiPAP)
Reference treatment: High Flow Nasal Cannula (HFNC)
Outcomes: Nasal injury

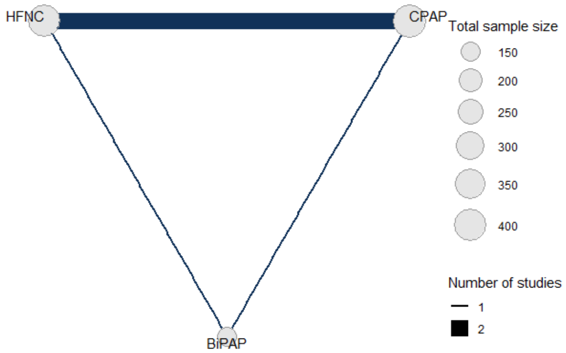

Outcome: Nasal injury. Rate in reference population: 1.8% (18 per 1000)

**Intervention, Total studies, Total participants**	**Odds ratio (95% Credible Interval, CrI)**	**Anticipated absolute effect (95% CrI)**			**Certainty of evidence**	**Ranking (SUCRA)**
		Without interventio n	***With intervention***	**Difference**		
HFNC<br>3 trials, 768 participants.	Reference	Reference	Reference	Reference	Reference	0.925
CPAP<br>3 trials, 852 participants	2.7 (0.84, 15)	18 per 1000	47 per 1000	29 more per 1000<br>(3 fewer to 180 more)	Very low[a]	0.319
BiPAP<br>2 trials, 284 participants	3.1 (0.78, 21)	18 per 1000	54 per 1000	36 more per 1000<br>(4 fewer to 250 more)	Very low[a]	0.254
Relative effects with reference to HFNC	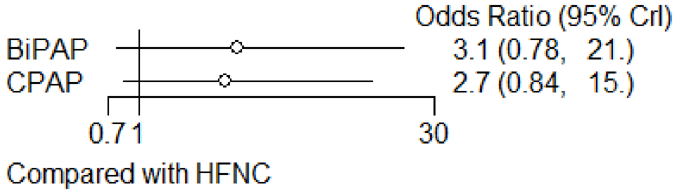					

[a]Downgraded due to serious risk of bias and serious imprecision.

Nasal injury, Effect estimates for all comparisons (Odds ratio, 95% CrI):

HFNC	CPAP	BiPAP
HFNC		
2.69 (0.8, 13) [b]	CPAP	
3.1 (0.80, 20) [b]	1.15 (0.26, 5.0) [b]	BiPAP

[b]Very low certainty of effect estimate

eTable 8. Summary of findings: Abdominal distension

© 2023 Iyer NP et al. *JAMA Pediatrics.*

Effects of estimates and certainty of evidence for post-extubation noninvasive respiratory support

Population: Critically ill children intubated and mechanically ventilated for at least 24 hours.
Setting: PICU, CICU
Interventions:
Continuous Positive Airway Pressure (CPAP)
Bi-Level Positive Airway Pressure (BiPAP)
Reference treatment: High Flow Nasal Cannula (HFNC)
Outcomes: Abdominal distension

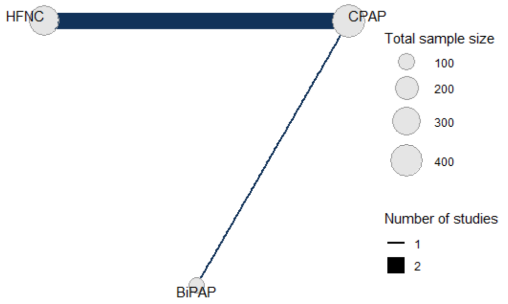

Outcome: Abdominal distension. Rate in reference population: 2.3% (23 per 1000)

**Intervention, Total studies, Total participants**	**Odds ratio (95% Credible Interval, CrI)**	**Anticipated absolute effect (95% CrI)**			**Certainty of evidence**	**Ranking (SUCRA)**
		Without intervention	***With intervention***	**Difference**		
HFNC<br>2 trials with non-zero events, 666 participants	Reference	Reference	Reference	Reference	Reference	0.446
CPAP<br>3 trials, 852 participants	0.99 (0.31, 3.3)	23 per 1000	23 per 1000	0 fewer per 1000 (16 fewer to 49 more)	Very low[a]	0.435
BiPAP<br>1 trial, 182 participants	0.78 (0.09, 6.5)	23 per 1000	18 per 1000	5 fewer per 1000 (21 fewer to 106 more)	Very low[a]	0.618
COT. 2 trials, 241 participants	Zero events	Zero events	Zero events	Zero events	-	-
Relative effects with reference to HFNC	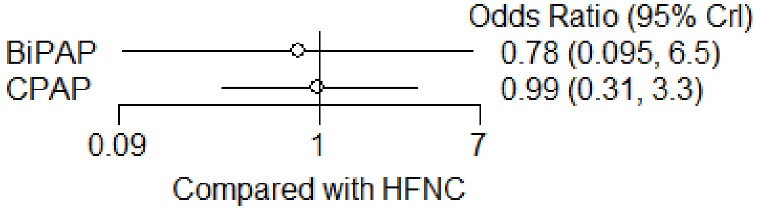					

[a]Downgraded due to serious risk of bias and serious imprecision.

Abdominal distension, Effect estimates of all comparisons (Odds ratio, 95% CrI):

HFNC	CPAP	BiPAP
HFNC		
0.99 (0.31, 3.25) [b]	CPAP	
0.78 (0.09, 6.5) [b]	1.25 (0.22, 7.37) [b]	BiPAP

[b]Very low certainty of effect estimate

eTable 9. Summary of findings for pair-wise analysis between CPAP and HFNC

© 2023 Iyer NP et al. *JAMA Pediatrics.*

CPAP compared to HFNC for post-extubation non-invasive respiratory support

Patient or population: post-extubation non-invasive respiratory support

Setting: PICU **Intervention:** CPAP **Comparison:** HFNC

Outcomes	№ of participants (studies) Follow-up	Certainty of the evidence (GRADE)	Relative effect (95% CI)	Anticipated absolute effects	
				Risk with HFNC	Risk difference with CPAP
Mortality, hospital	635 (2 RCTs)	⊕⊕◯◯ Low[a,c]	**OR 0.38** (0.15 to 0.97)	50 per 1,000	**30 fewer per 1,000** (42 fewer to 1 fewer)
Aspiration	666 (2 RCTs)	⊕◯◯◯ Very low[a,b]	**OR 1.00** (0.21 to 4.73)	9 per 1,000	**0 fewer per 1,000** (7 fewer to 32 more)
Sedation use	589 (2 RCTs)	⊕⊕◯◯ Low[a,b]	**OR 0.95** (0.83 to 1.09)	580 per 1,000	**13 fewer per 1,000** (46 fewer to 21 more)

***The risk in the intervention group** (and its 95% confidence interval) is based on the assumed risk in the comparison group and the **relative effect** of the intervention (and its 95% CI).
CI: confidence interval; **MD:** mean difference; **OR:** odds ratio

GRADE Working Group grades of evidence
High certainty: we are very confident that the true effect lies close to that of the estimate of the effect.
Moderate certainty: we are moderately confident in the effect estimate: the true effect is likely to be close to the estimate of the effect, but there is a possibility that it is substantially different.
Low certainty: our confidence in the effect estimate is limited: the true effect may be substantially different from the estimate of the effect.
Very low certainty: we have very little confidence in the effect estimate: the true effect is likely to be substantially different from the estimate of effect.

a. Lack of blinding and allocation not blinded to some participants. Crossover to other intervention permitted.

b. Wide 95% CI that includes benefit to either intervention

c. Wide 95% CI that includes lack of clinically meaningful benefit

eFigure 1. PRISMA Flow of information

© 2023 Iyer NP et al. *JAMA Pediatrics.*

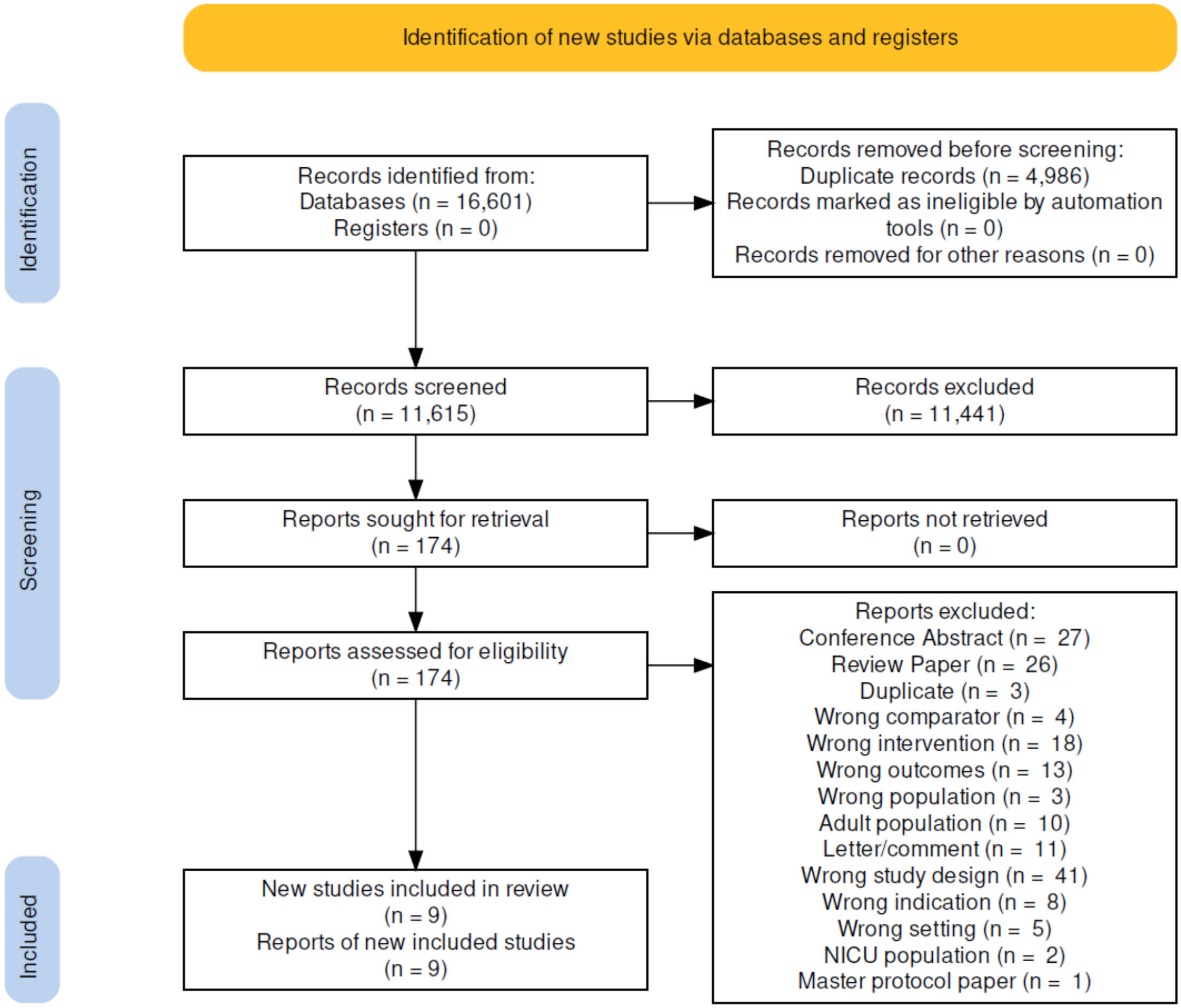

eFigure 2. Risk of bias. Extubation failure[8-16]

© 2023 Iyer NP et al. *JAMA Pediatrics.*

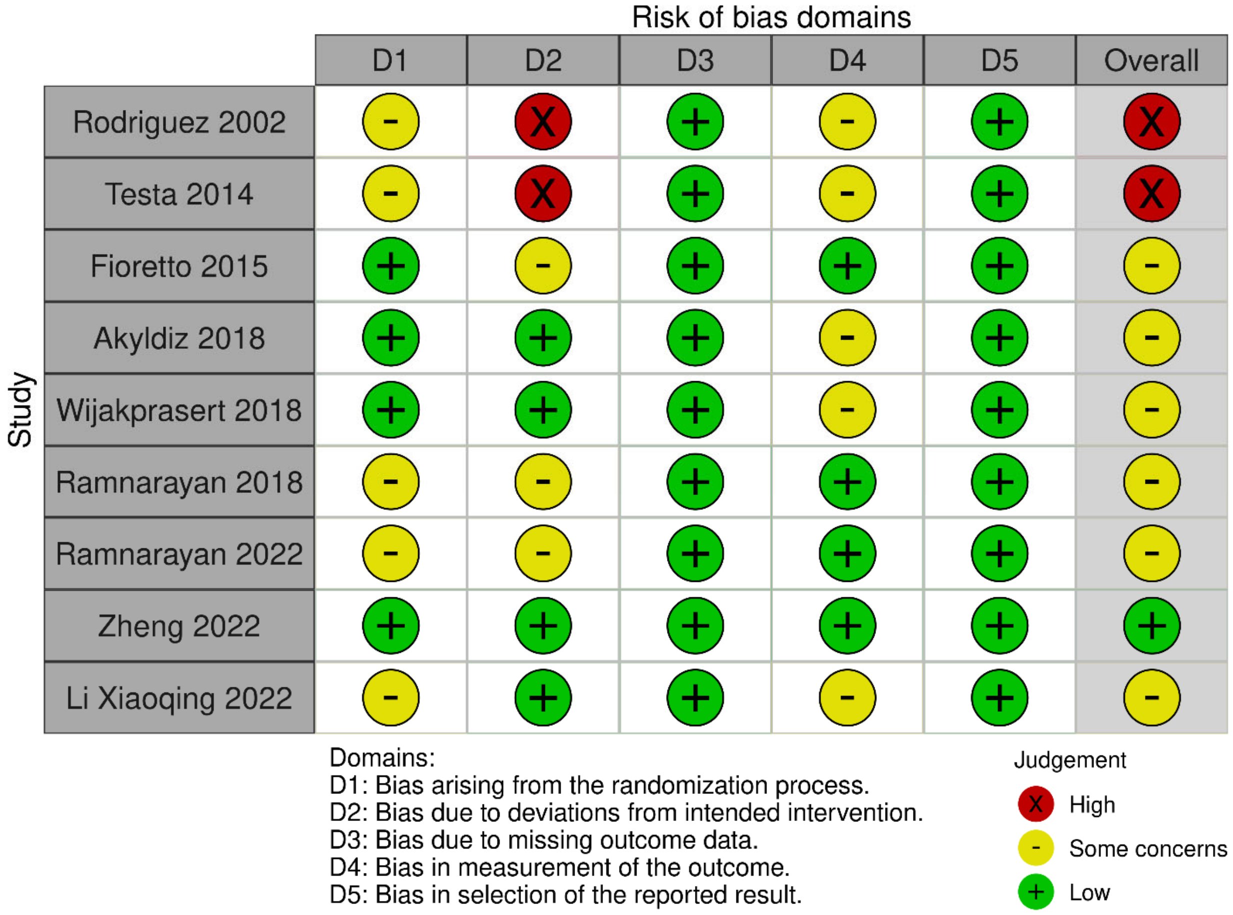

eFigure 3. Risk of bias: Treatment failure[8-16]

© 2023 Iyer NP et al. *JAMA Pediatrics.*

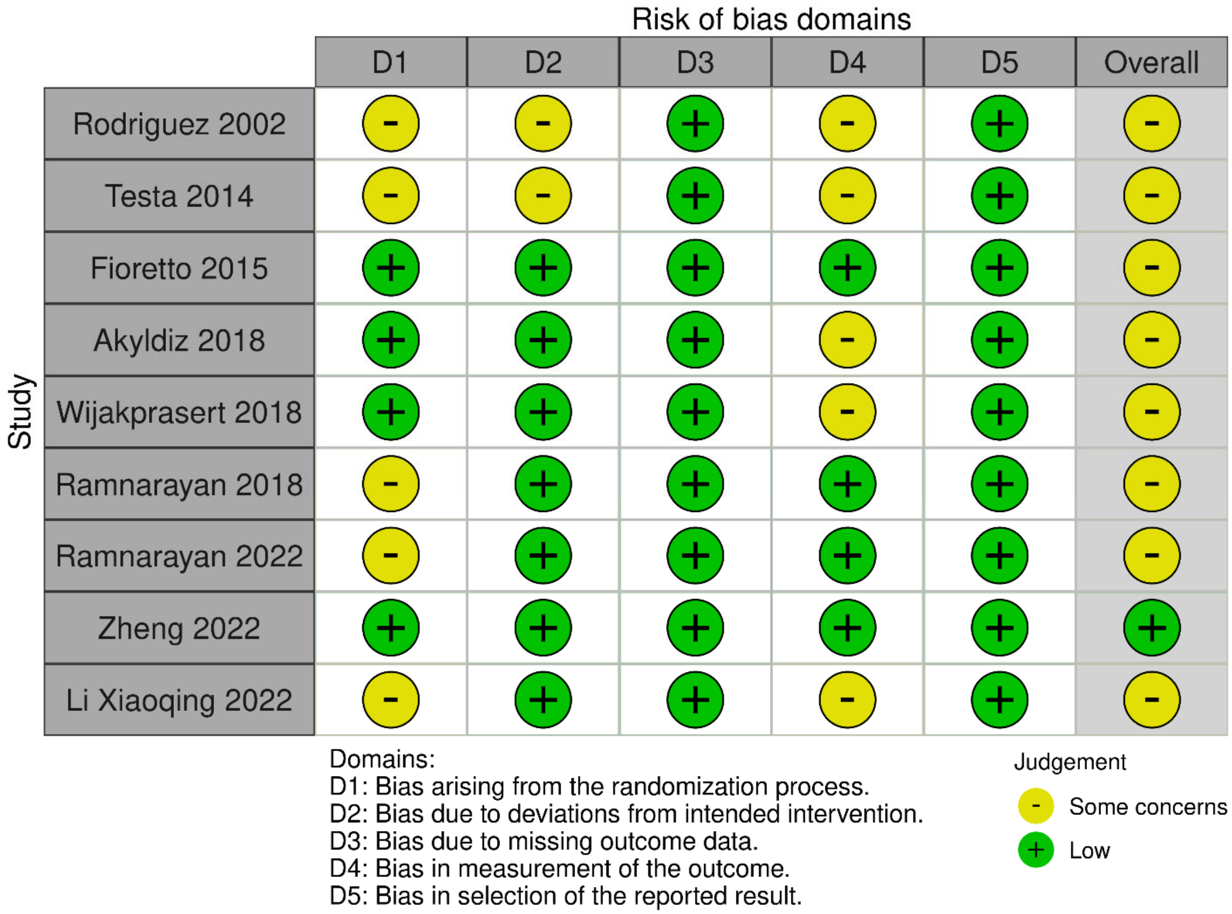

© 2023 Iyer NP et al. *JAMA Pediatrics.*

eFigure 4. Risk of bias: Abdominal distension[10,11,13,14,16]

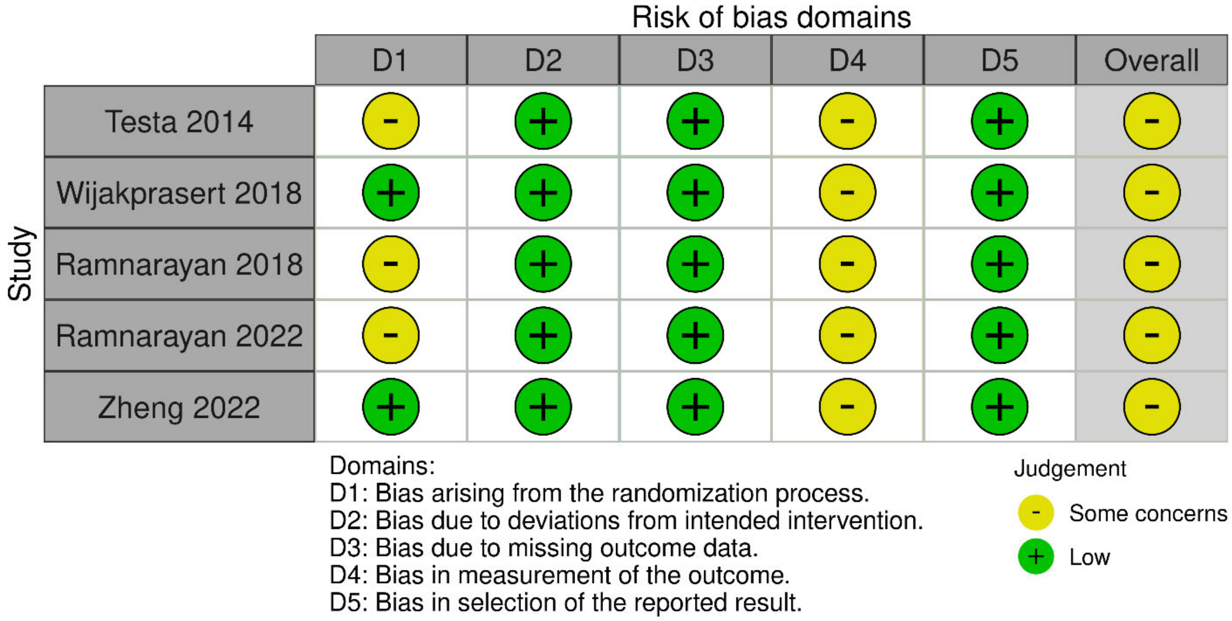

© 2023 Iyer NP et al. *JAMA Pediatrics.*

eFigure 5. Risk of bias: Hospital length of stay[10,11,14]

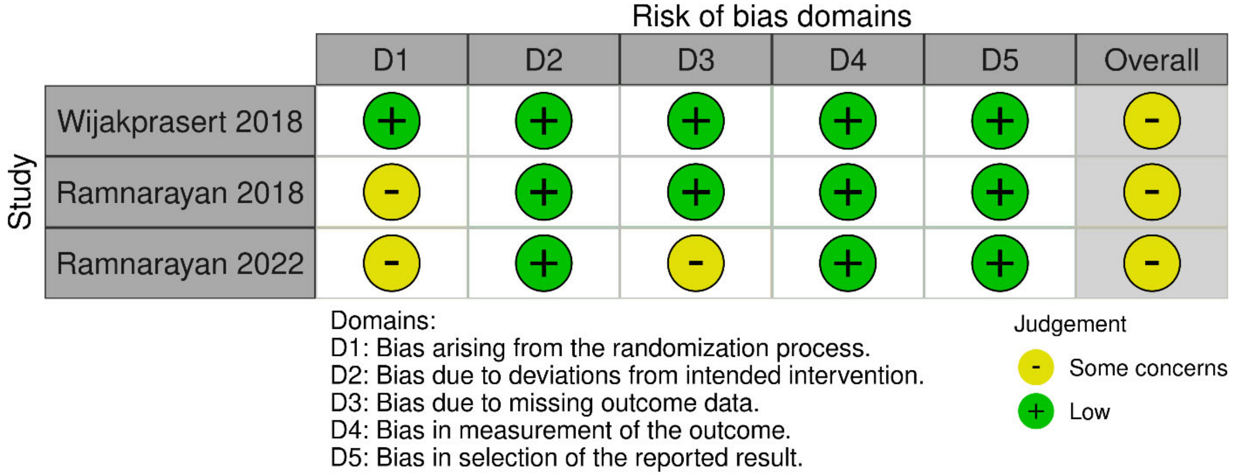

© 2023 Iyer NP et al. *JAMA Pediatrics.*

eFigure 6. Risk of bias: PICU mortality[10,11,14,15]

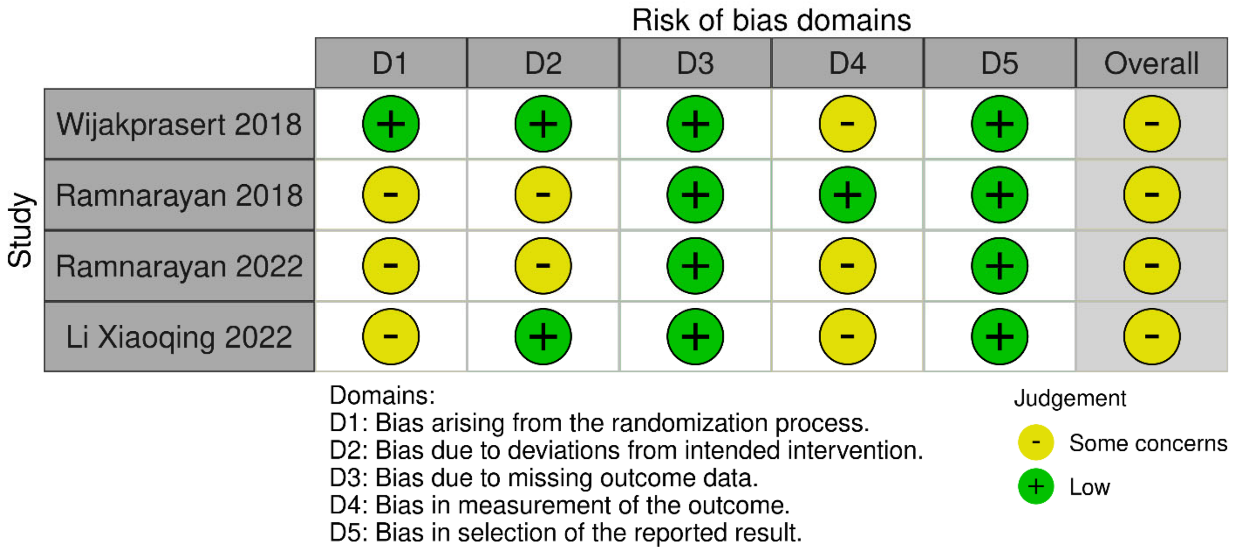

eFigure 7. Risk of bias: Nasal injury[10,11,13-16]

© 2023 Iyer NP et al. *JAMA Pediatrics.*

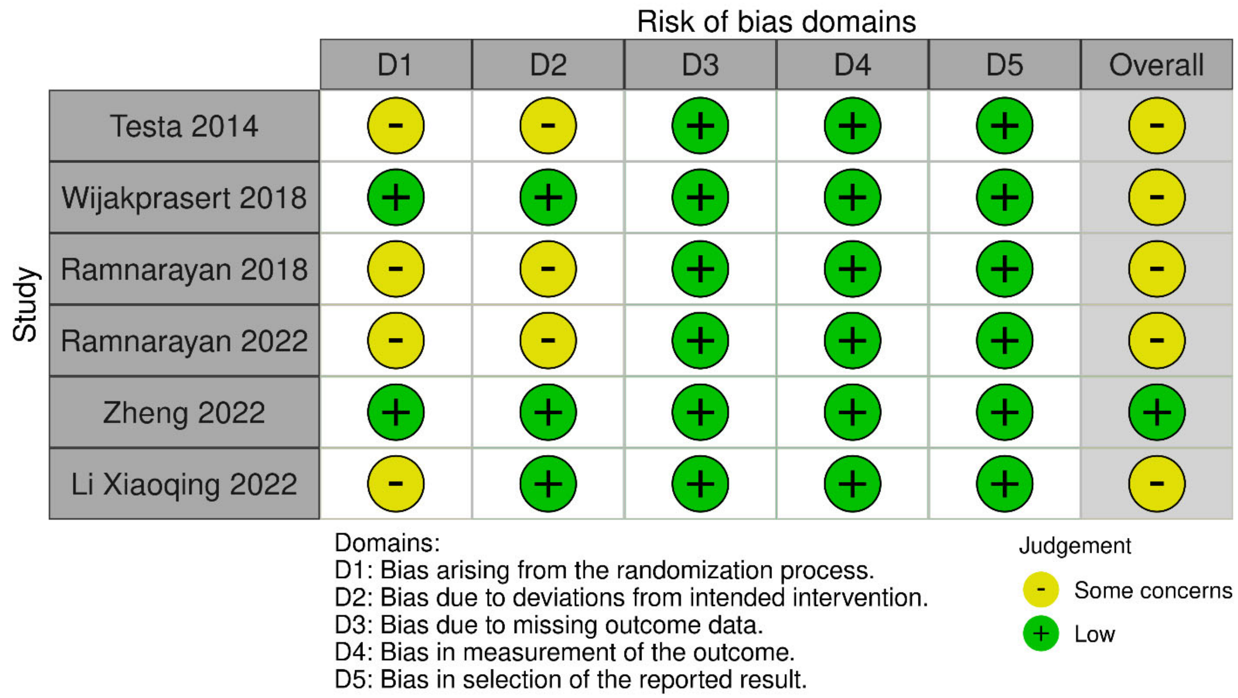

eFigure 8. Risk of bias: PICU LOS[10,11,13,14]

© 2023 Iyer NP et al. *JAMA Pediatrics.*

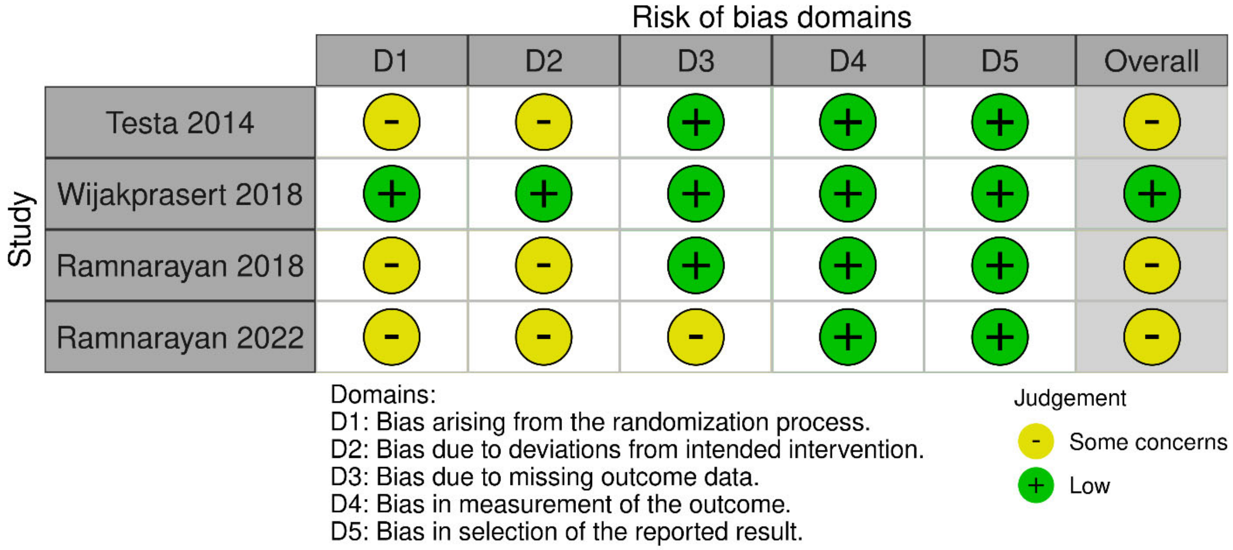

eFigure 9. Risk of bias: Hospital mortality[10,11]

© 2023 Iyer NP et al. *JAMA Pediatrics.*

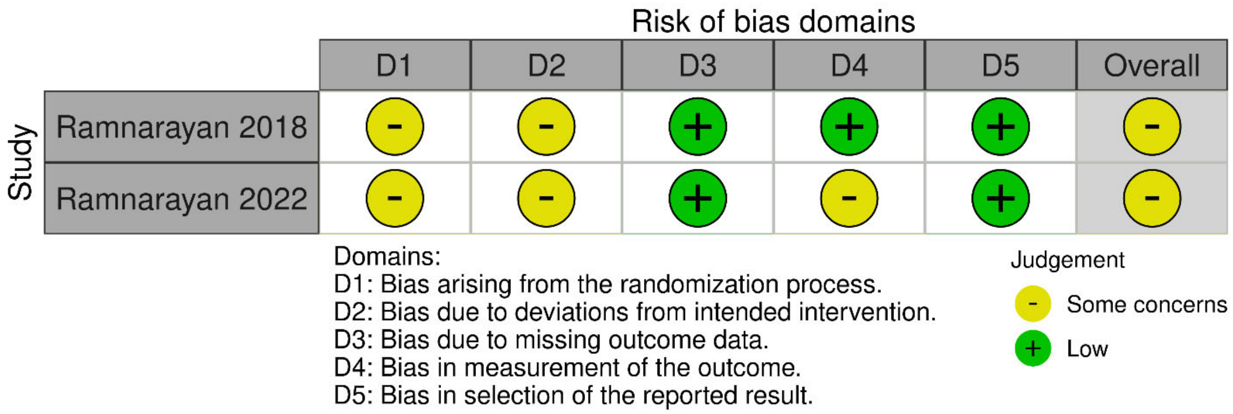

eFigure 10. Risk of bias: Sedation use[10,11]

© 2023 Iyer NP et al. *JAMA Pediatrics.*

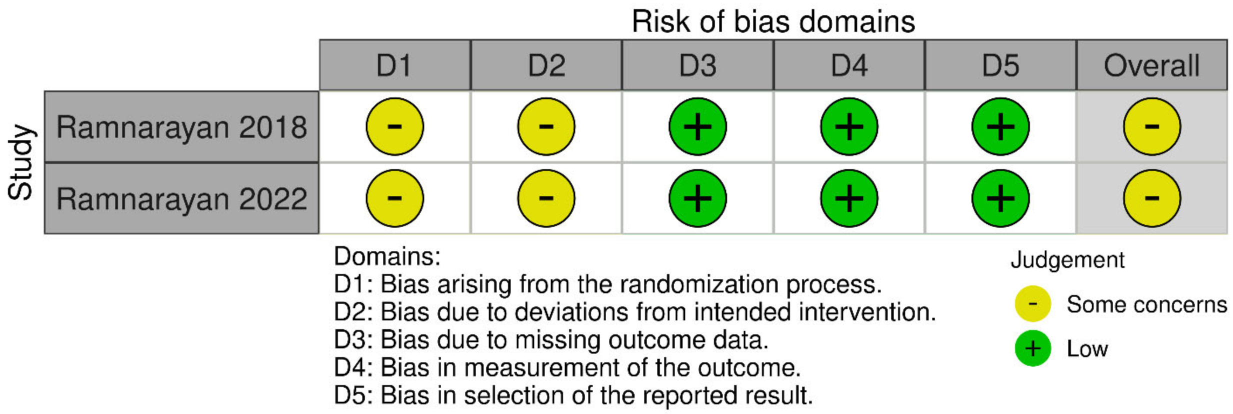

eFigure 11. Risk of bias: Aspiration[10,11]

© 2023 Iyer NP et al. *JAMA Pediatrics.*

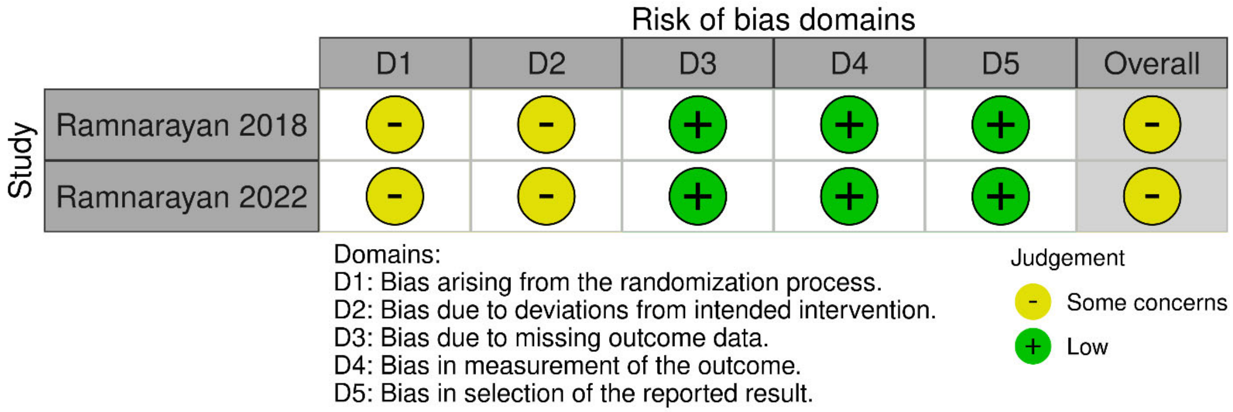

Figure 12a. Pairwise metanalysis: Forest plot of comparison: HFNC versus CPAP, outcome: Mortality, Hospital[10,11]

© 2023 Iyer NP et al. *JAMA Pediatrics.*

Study or Subgroup	CPAP Events	CPAP Total	HFNC Events	HFNC Total	Weight	Odds Ratio M-H, Fixed, 95% CI
Ramnarayan 2018	1	40	3	42	18.5%	0.33 [0.03, 3.35]
Ramnarayan 2022	5	272	13	281	81.5%	0.39 [0.14, 1.10]
**Total (95% CI)**		**312**		**323**	**100.0%**	**0.38 [0.15, 0.97]**
Total events	6		16			

Heterogeneity: Chi² = 0.01, df = 1 (P = 0.91); I² = 0%
Test for overall effect: Z = 2.01 (P = 0.04)

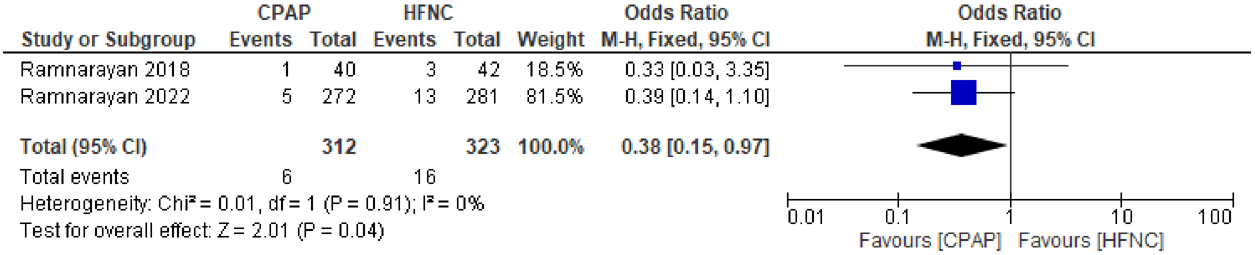

Figure 12b. Pairwise metanalysis: Forest plot of comparison: HFNC versus CPAP, outcome: Aspiration[10,11]

Study or Subgroup	CPAP Events	CPAP Total	HFNC Events	HFNC Total	Weight	Risk Ratio M-H, Random, 95% CI
Ramnarayan 2018	1	54	0	59	23.9%	3.27 [0.14, 78.67]
Ramnarayan 2022	2	272	3	281	76.1%	0.69 [0.12, 4.09]
**Total (95% CI)**		**326**		**340**	**100.0%**	**1.00 [0.21, 4.73]**
Total events	3		3			

Heterogeneity: Tau² = 0.00; Chi² = 0.71, df = 1 (P = 0.40); I² = 0%
Test for overall effect: Z = 0.00 (P = 1.00)

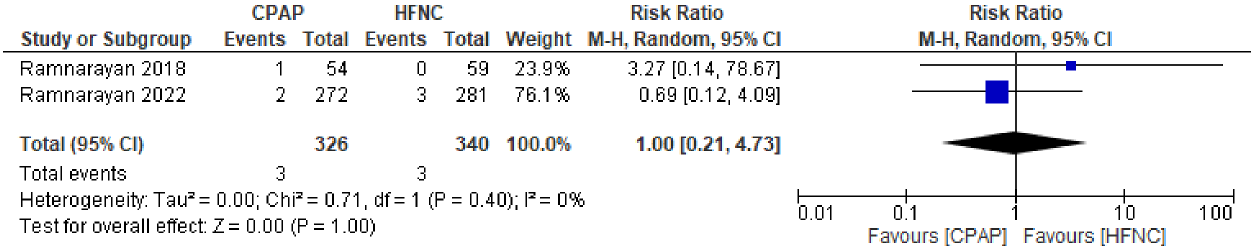

Figure 12c. Pairwise metanalysis: Forest plot of comparison: HFNC versus CPAP, outcome: Sedation use[10,11]

Study or Subgroup	CPAP Events	CPAP Total	HFNC Events	HFNC Total	Weight	Risk Ratio M-H, Random, 95% CI
Ramnarayan 2018	15	36	14	39	6.0%	1.16 [0.66, 2.05]
Ramnarayan 2022	141	246	164	268	94.0%	0.94 [0.81, 1.08]
**Total (95% CI)**		**282**		**307**	**100.0%**	**0.95 [0.83, 1.09]**
Total events	156		178			

Heterogeneity: Tau² = 0.00; Chi² = 0.52, df = 1 (P = 0.47); I² = 0%
Test for overall effect: Z = 0.74 (P = 0.46)

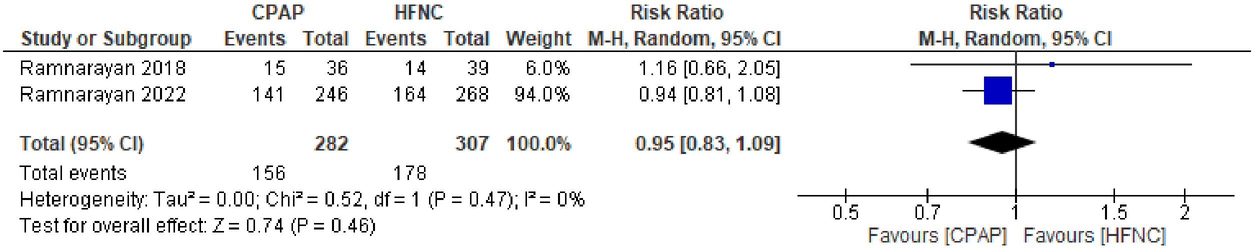

References:

© 2023 Iyer NP et al. *JAMA Pediatrics.*

1. Turner RM, Jackson D, Wei Y, Thompson SG, Higgins JP. Predictive distributions for between-study heterogeneity and simple methods for their application in Bayesian meta-analysis. *Stat Med.* 2015;34(6):984-998.
2. Dias S, Sutton AJ, Ades AE, Welton NJ. Evidence synthesis for decision making 2: a generalized linear modeling framework for pairwise and network meta-analysis of randomized controlled trials. *Med Decis Making.* 2013;33(5):607-617.
3. Brooks SP, Gelman A. General Methods for Monitoring Convergence of Iterative Simulations. *Journal of Computational and Graphical Statistics.* 1998;7(4):434-455.
4. Dias S, Welton NJ, Sutton AJ, Caldwell DM, Lu G, Ades AE. Evidence synthesis for decision making 4: inconsistency in networks of evidence based on randomized controlled trials. *Med Decis Making.* 2013;33(5):641-656.
5. Brignardello-Petersen R, Bonner A, Alexander PE, et al. Advances in the GRADE approach to rate the certainty in estimates from a network meta-analysis. *J Clin Epidemiol.* 2018;93:36-44.
6. Brignardello-Petersen R, Mustafa RA, Siemieniuk RAC, et al. GRADE approach to rate the certainty from a network meta-analysis: addressing incoherence. *J Clin Epidemiol.* 2019;108:77-85.
7. Hultcrantz M, Rind D, Akl EA, et al. The GRADE Working Group clarifies the construct of certainty of evidence. *J Clin Epidemiol.* 2017;87:4-13.
8. Akyıldız B, Öztürk S, Ülgen-Tekerek N, Doğanay S, Görkem SB. Comparison between high-flow nasal oxygen cannula and conventional oxygen therapy after extubation in pediatric intensive care unit. *Turk J Pediatr.* 2018;60(2):126-133.
9. Fioretto JR, Ribeiro CF, Carpi MF, et al. Comparison between noninvasive mechanical ventilation and standard oxygen therapy in children up to 3 years old with respiratory failure after extubation: a pilot prospective randomized clinical study. *Pediatr Crit Care Med.* 2015;16(2):124-130.
10. Ramnarayan P, Lister P, Dominguez T, et al. FIRST-line support for Assistance in Breathing in Children (FIRST-ABC): a multicentre pilot randomised controlled trial of high-flow nasal cannula therapy versus continuous positive airway pressure in paediatric critical care. *Crit Care.* 2018;22(1):144.
11. Ramnarayan P, Richards-Belle A, Drikite L, et al. Effect of High-Flow Nasal Cannula Therapy vs Continuous Positive Airway Pressure Following Extubation on Liberation From Respiratory Support in Critically Ill Children: A Randomized Clinical Trial. *JAMA.* 2022;327(16):1555-1565.
12. Rodriguez JA, Von Dessauer B, Duffau G. [Non-invasive continuous positive airways pressure for post-extubation laryngitis in pediatric patients]. *Archivos de bronconeumologia.* 2002;38(10):463-467.
13. Testa G, Iodice F, Ricci Z, et al. Comparative evaluation of high-flow nasal cannula and conventional oxygen therapy in paediatric cardiac surgical patients: a randomized controlled trial. *Interact Cardiovasc Thorac Surg.* 2014;19(3):456-461.
14. Wijakprasert P, Chomchoey J. High-flow nasal cannula versus conventional oxygen therapy in post-extubation pediatric patients: A randomized controlled trial. *Journal of the Medical Association of Thailand.* 2018;101(10):1331-1335.
15. Li XQ, Zhao WL, Li DY, Lei L, Luo LL, Qiao LN. Clinical Study on Early Extubation and Sequential Non-Invasive Respiratory Support for Children with Acute Respiratory Failure Receiving Invasive Mechanical Ventilation. *Sichuan da xue xue bao Yi xue ban = Journal of Sichuan University Medical science edition.* 2022;53(2):321-326.
16. Zheng YR, Lin WH, Lin SH, Xu N, Cao H, Chen Q. Bi-level Positive Airway Pressure Versus Nasal CPAP for the Prevention of Extubation Failure in Infants After Cardiac Surgery. *Respir Care.* 2022;67(4):448-454.

© 2023 Iyer NP et al. *JAMA Pediatrics.*

© 2023 Iyer NP et al. *JAMA Pediatrics.*
