# Supplementary material for: Association of Extubation Failure Rates With High-Flow Nasal Cannula, Continuous Positive Airway Pressure, and Bilevel Positive Airway Pressure vs Conventional Oxygen Therapy in Infants and Young Children: A Systematic Review and Network Meta-Analysis
Source: JAMA Pediatr. 2023 Jun 5;177(8):774–81. doi: 10.1001/jamapediatrics.2023.1478 (PMC10242512; doi:10.1001/jamapediatrics.2023.1478)
Supplement: Supplement 2. — Data sharing statement [file jamapediatr-e231478-s002.pdf]

Data Sharing Statement

Iyer. Association of Extubation Failure Rates With High-Flow Nasal Cannula, Continuous Positive Airway Pressure, and Bilevel Positive Airway Pressure vs Conventional Oxygen Therapy in Infants and Young Children. *JAMA Pediatr*. Published June 05, 2023. doi:10.1001/jamapediatrics.2023.1478

Data
Data available: No

Additional Information
Explanation for why data not available: Data for this study has been extracted from previously published randomized trials which are already publicly available.
